# Neuromarketing and the gaming consumer: a systematic review of attention, emotion, and memory in player engagement and retention

**DOI:** 10.3389/fnrgo.2026.1918889

**Published:** 2026-09-10

**Authors:** Sandra Jimenez-Duarte, Guillermo Castilla-Cebrian

**Affiliations:** 1 Facultad de Diseno y Tecnologias Creativas, Universidad Europea de Madrid, Madrid, Spain; 2 School of Architecture, Engineering, Science and Computing - STEAM, Universidad Europea de Madrid, Villaviciosa de Odon, Spain

**Keywords:** consumer behavior, digital marketing, emotional retention, neuromarketing, PRISMA, user engagement, video games

## Abstract

The global video game industry has overtaken other cultural sectors in scale, intensifying competition for player attention, engagement, and long-term retention. Increasingly, firms pair digital marketing with neuromarketing, a set of methods drawn from cognitive neuroscience that measure attention, emotion, and memory to understand the implicit processes underlying consumer decisions. Yet evidence on how these approaches shape retention in gaming, and on how their effects can be validly measured, remains fragmented. This systematic review, following PRISMA 2020, synthesizes 54 studies published between 2009 and 2025, identified through Boolean searching of three bibliographic databases and supplemented by citation tracking. Studies were eligible if they addressed this intersection in video game contexts, or in adjacent consumer settings serving as methodological models; each was appraised for risk of bias by two reviewers independently, using AMSTAR 2, JBI and CASP instruments according to design, and common criteria of sampling clarity, procedural transparency and replicability for quantitative studies. The corpus combined quantitative-empirical (64.8%), mixed or theoretical (25.9%), and qualitative (9.3%) designs, of which 51.9% were rated high quality. Two complementary measurement streams emerged: behavioral and self-report approaches, and physiological or neuromarketing approaches using electroencephalography, eye-tracking, galvanic skin response, and facial coding. Across studies, implicit indices of attention allocation, affective response, and memory encoding were associated with engagement and with antecedents of retention beyond self-report. Studies reporting attention and engagement outcomes more often favored neuromarketing-informed approaches, but this evidence is largely associational rather than based on direct comparison with conventional campaigns, and reported magnitudes varied too widely to pool. Convergent evidence linked visual congruence, narrative and emotional design, parasocial influencer relationships, community co-creation, and algorithmic personalization to sustained play. Three limitations recur: a reliance on cross-sectional rather than longitudinal designs, the limited ecological validity of laboratory measurement, and a geographic concentration in Western Europe, North America, and East Asia. We further distinguish static neuromarketing measurement from dynamic, neuromarketing-informed design, arguing that the latter raises distinct ethical concerns for autonomy, biometric data, and vulnerable players. The review offers an integrative framework for studying the gaming consumer and sets out priorities for ecologically valid, ethically governed research.

## 1 Introduction

The video game sector has undergone a profound transformation since its beginnings as a form of recreational entertainment in the final decades of the 20th century. Over the years, video games have evolved from simple digital pastimes to become platforms for socialization, spaces for cultural expression, and environments for economic interaction with a significant global impact. This evolution has been driven by technological factors such as advancements in graphic engines, the development of multi-platform consoles, and, especially, the widespread access to the internet, which has facilitated the emergence of multiplayer experiences, virtual communities, and burgeoning digital economies.

According to the Newzoo (2023), the global video game market now exceeds 200 billion US dollars annually, positioning itself ahead of other cultural industries such as cinema and music. More recent industry analyses project continued growth driven by mobile gaming expansion and the emergence of cloud-based subscription models, with MENA and LATAM regions recording the strongest install growth rates in 2024 (Adjust, 2025). This expansion has fostered not only a diversification of formats and content but also a growing sophistication in marketing strategies aimed at attracting and retaining an increasingly diverse and globalized audience. Today's video game consumer no longer fits a homogeneous profile but instead encompasses multiple age, sociocultural, and behavioral segments. This diversity has compelled companies to develop highly personalized strategies based on interaction, emotional engagement, and digital identity.

In parallel, emerging disciplines such as neuromarketing have begun to integrate into this ecosystem with the aim of gaining deeper insight into the underlying motivations of consumers. By combining neuroscience, cognitive psychology, and data analytics, these tools enable the design of more effective brand experiences, measuring with greater precision variables such as attention, emotion, and memory. In this context, it becomes essential to understand how digital marketing strategies and neurocognitive technologies converge in shaping the experience of the contemporary gamer.

### 1.1 Justification and objectives of the systematic review

Despite the growing academic and business interest in the impact of digital marketing and neuromarketing on the video game sector, there remains considerable fragmentation in the literature. Numerous empirical studies exist on the use of influencers, social networks, and emotional strategies in video game advertising campaigns; likewise, isolated aspects of neuroscience applied to digital consumption (such as visual attention, emotion elicitation, or reward processing) have been explored. However, a systematic review that articulates these approaches transversally, evaluating both marketing strategies and consumer responses within a unified methodological framework, is still lacking.

This review addresses four interconnected needs in the literature. First, the field lacks an integrative comparison of digital marketing strategies aimed at consumer retention across gaming platforms. Second, the effectiveness of neuromarketing as a tool for experience design has been assessed piecemeal rather than systematically. Third, the analytical methodologies employed to measure emotions, attention, engagement, and loyalty have not been evaluated against each other within a unified framework. Fourth, the intersection of marketing, neuroscience, and video games remains undertheorized, leaving practical gaps in how industry actors translate neurocognitive insights into actionable campaign design. A recent systematic review by Gupta et al. (2025) confirms that while neuromarketing tools have been widely applied to pre-purchase decision stages, their role across the full consumer lifecycle, including post-purchase retention, remains underexplored, a gap the present review is specifically positioned to address.

Furthermore, this review is framed within a context of growing demand for interdisciplinary approaches that can explain complex phenomena of digital consumption from multiple levels: technological, emotional, social, and cognitive.

### 1.2 Research question

How do digital marketing strategies and neuromarketing influence video game consumer retention, and what methodological approaches allow this influence to be evaluated comprehensively and with empirical validation?

Because this review synthesizes evidence across several closely related but non-equivalent constructs, we define them at the outset and maintain the distinction throughout. Attention refers to the allocation of perceptual and cognitive resources to a stimulus; emotion/affect refers to valence- and arousal-based responses; memory refers to the encoding, retention, and retrieval of marketing- or game-related information; engagement refers to a multidimensional state of involvement expressed behaviorally (e.g., session length), cognitively, and affectively during exposure; and retention refers to sustained, repeated use of a game or platform over time. Attention, emotion, memory, and engagement are treated here as potential antecedents or proxies of retention rather than as retention itself: only studies with longitudinal or repeated-behavior designs measured retention directly, whereas most studies measured immediate or cross-sectional outcomes from which continued use is inferred. Throughout, we reserve causal wording for studies whose designs support it and use associational language (“was associated with”, “may contribute to”) elsewhere.

Eligibility criteria and the review protocol are set out in Section 2.

In terms of consumer behavior analysis, the funnel diagram emerges as a key tool. This model, based on the stages of awareness-consideration-conversion, enables the optimization of strategic decisions through data analysis (Keller and Kotler, 2006; Saura et al., 2020). Its usefulness is reinforced by methodologies such as:

- Cohort analysis
- Heatmaps
- Attribution models

These techniques help capture friction points and user motivators more accurately throughout the gamer consumer's lifecycle.

## 2 Methods

To ensure the scientific rigor of this systematic review, a methodological strategy was adopted based on the recommendations of the PRISMA 2020 protocol (Preferred Reporting Items for Systematic Reviews and Meta-Analyses) (Moher et al., 2009; Page et al., 2021). This protocol provides a standardized framework for developing reviews that are transparent, reproducible, and comprehensive, including the following steps: (1) stating the rationale motivating the review and clearly formulating the research question; (2) defining inclusion criteria and the bibliographic sources to be consulted; (3) detailing the search strategy and the procedure for selecting studies; (4) describing the data extraction methods, outcomes of interest, and techniques used to assess risk of bias; and (5) presenting the results accompanied by a detailed explanation of the entire review process. This approach ensures adequate coverage of the most relevant sources, as well as an objective selection of the included studies.

The review focused on empirical studies, previous reviews, theoretical articles, and methodological contributions addressing the intersection of digital marketing, neuromarketing, and consumer behavior in the context of video games. To this end, a structured search protocol was implemented in high-impact scientific databases, applying rigorous selection and exclusion criteria.

### 2.1 Development of the search protocol

The literature search was conducted between 29 April and 2 May 2025. Records were identified through two complementary routes. The first was systematic Boolean searching of three bibliographic databases, selected for their coverage of applied social sciences, marketing, and behavioral research.

The first route was systematic Boolean searching of three bibliographic databases: the Web of Science Core Collection (searched 29 April 2025; five queries; 381 records), Scopus (29 April 2025; four queries; 297 records), and the Psychology and Behavioral Sciences Collection via EBSCOhost (2 May 2025; four queries; 654 records). Because each platform applies its own indexing vocabulary and operator syntax, queries were adapted to the field structure of each database rather than transferred verbatim. Because each platform applies its own indexing vocabulary and operator syntax, queries were adapted to the field structure of each database rather than transferred verbatim. The queries combined four concept blocks, namely consumer role (consumer, customer, prosumer), platform and channel (social media, Instagram), audiovisual format (audiovisual, video, reel), and commercial content (advertisement, commercial), with video game terminology applied as a fifth block in those queries directed at the gaming literature. The complete strings as executed, together with the number of records returned by each, are reported in Supplementary Appendix S1.

**Figure 1:**
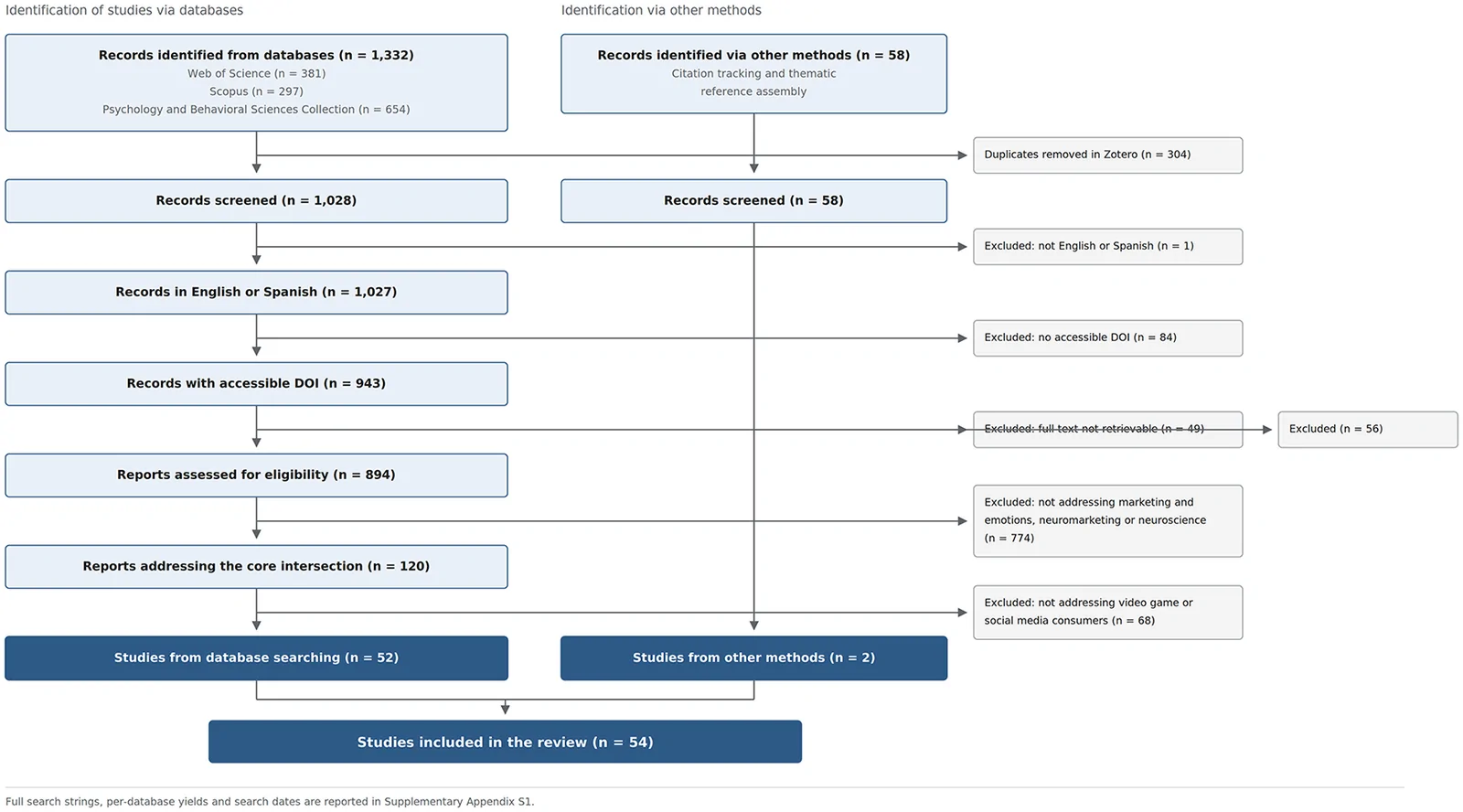
PRISMA diagram.

The database queries returned 1,332 records. Records were exported in RIS format and imported into Zotero (version 7.x), where 304 duplicates were identified through automated detection and verified manually before removal, leaving 1,028 unique records for screening. The screening cascade from 1,028 records to the final sample of 54 is reported in Figure 1.

The second identification route was purposive and citation-based. The Boolean queries above address the digital marketing and video game literature and do not incorporate neuromarketing or psychophysiological method terminology; of the 1,028 unique records, only one refers to neuromarketing in its title or abstract. The conceptual and neuromarketing literature synthesized in this review was therefore identified separately, through reference-list tracking of included studies and through a thematically assembled set of foundational sources, several of which are monographs or Spanish-language works not indexed in the databases searched. A supplementary forward-citation search was carried out in May 2025 using Google Scholar together with direct journal alerts for the five journals most frequently represented in the initial sample: Journal of Business Research, Psychology and Marketing, Frontiers in Psychology, Computers in Human Behavior, and Journal of Interactive Marketing. This two-route design was adopted deliberately, since the intersection examined here is not served by any single controlled vocabulary. It also accounts for part of the methodological heterogeneity discussed in Section 4.10.

The search criteria included:

- Languages: Spanish and English.
- Period: articles published between 2009 and 2025.
- Document types: peer-reviewed articles, doctoral dissertations, systematic reviews, and empirical studies. Monographs were retained only where they constitute foundational conceptual sources.
- Subject areas: marketing, consumer psychology, applied neuroscience, communication sciences, video games, and technology.

One characteristic of the executed search should be recorded. In the Psychology and Behavioral Sciences Collection, the third query was entered without parentheses around its OR operator and was consequently evaluated by the platform as a disjunction rather than as the intended conjunction, returning 615 of the 1,332 records. These records were subject to the same screening criteria as all others and the large majority were excluded at the title and abstract stage. The query is reported as executed in Supplementary Appendix S1 rather than corrected retrospectively.

### 2.2 Analysis and screening

The analysis of the identified documents was carried out in three phases:

***Phase 1: title and abstract screening***

Two researchers independently reviewed the titles and abstracts of the retrieved articles. Duplicates were removed, and studies unrelated to the research objective (e.g., investigations focused solely on technical video game design without consumer focus) were excluded.

Eligibility was specified using the PICOS framework (Table 1). The population of interest was players and digital consumers in video game or game-adjacent contexts, of any age, region or player type. The intervention or exposure was digital marketing strategy, neuromarketing, or psychophysiological measurement applied to consumer or player response; at least one had to be present, and explicit neuromarketing application was sufficient on its own. No comparator was required, since the field contains few controlled comparisons; where comparators were present they took the form of conventional campaigns, alternative stimuli or contrasting participant groups, and studies without a comparator were retained and are identified as such in Supplementary Table 2. Eligible outcomes were attention, emotion or affect, memory, engagement, purchase intention or spending, trust and brand attitude, and retention, measured by perceptual, behavioral or physiological means. Eligible designs were empirical studies of any type together with systematic reviews and theoretical or conceptual papers. The findings organized by PICOS element are reported in Section 3.

**Table 1:** Eligibility criteria specified using the PICOS framework.

PICOS element	Eligibility criterion applied
P (Population)	Players and digital consumers in video game or game-adjacent contexts, of any age, region or player type. Studies of general consumer populations were eligible only where they served as a methodological model for gaming contexts.
I (Intervention)	Digital marketing strategy, neuromarketing, or psychophysiological measurement applied to consumer or player response. At least one required; explicit neuromarketing application sufficient alone.
C (Comparator)	Not required. Where present: conventional campaigns, alternative stimuli or designs, or contrasting participant groups. Studies without a comparator were retained and identified as such.
O (Outcomes)	At least one of: attention, emotion or affect, memory, engagement, purchase intention or spending, trust and brand attitude, retention. Perceptual, behavioral and physiological measures all admissible.
S (Study design)	Empirical studies of any design, systematic reviews, and theoretical or conceptual papers. Peer-reviewed articles, doctoral dissertations and foundational monographs, published 2009 to 2025 in English or Spanish.

***Phase 2: Full-text reading and application of inclusion/exclusion criteria***

Articles were selected for full-text reading if they met the first criterion as mandatory (application of digital marketing techniques in video game contexts OR explicit neuromarketing application) plus at least one further criterion from the following:

- Application of digital marketing techniques in video game contexts.
- Studies on the emotional, cognitive, or visual behavior of gamer consumers.
- Explicit application or mention of neuromarketing.
- Measurable outcomes on engagement, retention, or decision-making.

A small number of studies from adjacent domains were retained under the mandatory criterion of explicit neuromarketing application, even where the population studied was not composed of players. These include virtual-retail eye-tracking work retained as a methodological model for visual-attention analysis, and survey research on practitioners' adoption of gamification and neuromarketing retained for its bearing on how these techniques enter commercial practice. Admitting such studies broadens the corpus beyond gaming populations and contributes to the heterogeneity discussed in Section 4.10. To make that consequence visible rather than implicit, every study is classified by ecological proximity to gaming in Section 3.1, and Table 2 reports the direction of findings separately for each class, so that a reader can distinguish conclusions resting on gaming-specific evidence from those that do not.

Table 2Direction of reported associations by outcome family, stratified by appraised study quality.

**Table:** 

Outcome family	High				Moderate				Low				Total
	+	0	±	–	+	0	±	–	+	0	±	–	
Panel A—by appraised quality													
A. Attention (visual allocation)	3	–	1	–	–	–	–	–	–	–	–	–	4
B. Arousal/affective response	2	–	–	–	–	–	–	–	–	–	–	–	2
C. Memory (recall/recognition)	1	–	1	–	–	–	–	–	–	–	–	–	2
D. Engagement (during exposure)	7	–	2	1	1	–	1	–	–	–	–	1	13
E. Purchase intention/spending	7	–	3	–	1	–	–	–	1	–	–	–	12
F. Trust, brand attitude and loyalty	9	–	1	1	2	–	–	–	2	–	–	–	15
G. Retention/continued use (over time)	4	–	–	1	–	–	–	–	1	–	–	–	6
Column total	33	–	8	3	4	–	1	–	4	–	–	1	54

**Table:** 

Outcome family	i				ii				iii				iv				Total
	+	0	±	–	+	0	±	–	+	0	±	–	+	0	±	–	
Panel B—by ecological class																	
A. Attention (visual allocation)	2	–	–	–	1	–	1	–	–	–	–	–	–	–	–	–	4
B. Arousal/affective response	2	–	–	–	–	–	–	–	–	–	–	–	–	–	–	–	2
C. Memory (recall/recognition)	1	–	–	–	–	–	1	–	–	–	–	–	–	–	–	–	2
D. Engagement (during exposure)	6	–	2	1	1	–	1	–	–	–	–	1	1	–	–	–	13
E. Purchase intention/spending	7	–	2	–	1	–	–	–	–	–	–	–	1	–	1	–	12
F. Trust, brand attitude and loyalty	12	–	1	–	1	–	–	1	–	–	–	–	–	–	–	–	15
G. Retention/continued use (over time)	–	–	–	–	3	–	–	1	–	–	–	–	2	–	–	–	6
Column total	30	–	5	1	7	–	3	2	–	–	–	1	4	–	1	–	54

Counts are numbers of studies, not effect sizes. + = association in the hypothesized direction; 0 = no reliable association; ± = mixed or moderator-dependent; – = association opposite to the hypothesized direction. A study contributing findings to more than one outcome family appears in each relevant row but only once within any row, so row totals exceed the number of included studies. Systematic reviews, meta-analyses, and theoretical papers are excluded from these counts to avoid counting their primary studies twice. No pooled effect estimate is reported. Direction-of-effect coding covers the 50 included studies reporting primary outcome data (35 quantitative, 5 qualitative, and 10 mixed-method or content-analytic studies). The remaining four studies, comprising two systematic reviews, one meta-analysis and one policy statement, report no primary outcome data and are excluded to avoid counting their constituent studies twice (50 + 4 = 54).

I, general advertising / retail (non-gaming); ii, game-related advertising; iii, actual gameplay; iv, field / longitudinal.

Exclusion criteria were:

- Documents without peer review.
- Studies focused solely on programming or technical development without a marketing dimension.
- Opinion pieces or essays lacking clear empirical or methodological support.

***Phase 3: coding and thematic analysis***

Once the final articles were selected, thematic coding was carried out using qualitative analysis based on emerging categories. The main categories were:

- Digital marketing strategies (segmentation, personalization, content).
- Emotions and decision-making.
- Neuromarketing techniques used (EEG, eye tracking, fMRI, etc.).
- Consumer retention and engagement.
- Influence of digital platforms and influencers.

This analysis enabled the identification of patterns, trends, and theoretical gaps that will be developed in subsequent sections. The methodological flow of this systematic review is illustrated in Figure 1.

After applying the full screening protocol, 120 potentially relevant studies remained. A final thematic refinement step, restricting inclusion to studies explicitly addressing the intersection of digital marketing, neuromarketing, and consumer behavior in video game contexts, reduced the sample to 54 highly relevant studies.

To maintain consistency and reduce subjectivity, all phases of the screening process were conducted by two reviewers, who divided the records between them. A random subsample of 220 records, representing 21.4% of the 1,028 unique records, was screened independently by both reviewers at the title-and-abstract stage to assess inter-rater reliability; agreement was 96.4% (212 of 220 records), with 8 disagreements, giving a Cohen's kappa of 0.82. Across the full screening process, including full-text assessment, 36 disagreements arose in total, all of which were resolved by discussion. The protocol provided for arbitration by a third senior researcher from the same research group had consensus not been reached, but this provision was not invoked. This procedure minimized selection bias.

The search and selection process was conducted using a structured recording sheet to ensure thematic coding consistency and traceability.

Supplementary Table 2, provides a structured summary of the studies included in the systematic review (*n* = 54), organized according to the methodological criteria established in the PRISMA 2020 protocol. For each study it reports the title, authors, methodological design, sample size, focus of findings, country, risk-of-bias rating, year of publication and identification route.

This table serves a dual purpose: on the one hand, it ensures transparency in the selection process and critical appraisal of included studies; on the other, it allows visualization of the geographical, theoretical, and methodological diversity that characterizes the field.

#### 2.2.1 Temporal distribution

**Figure 2:**
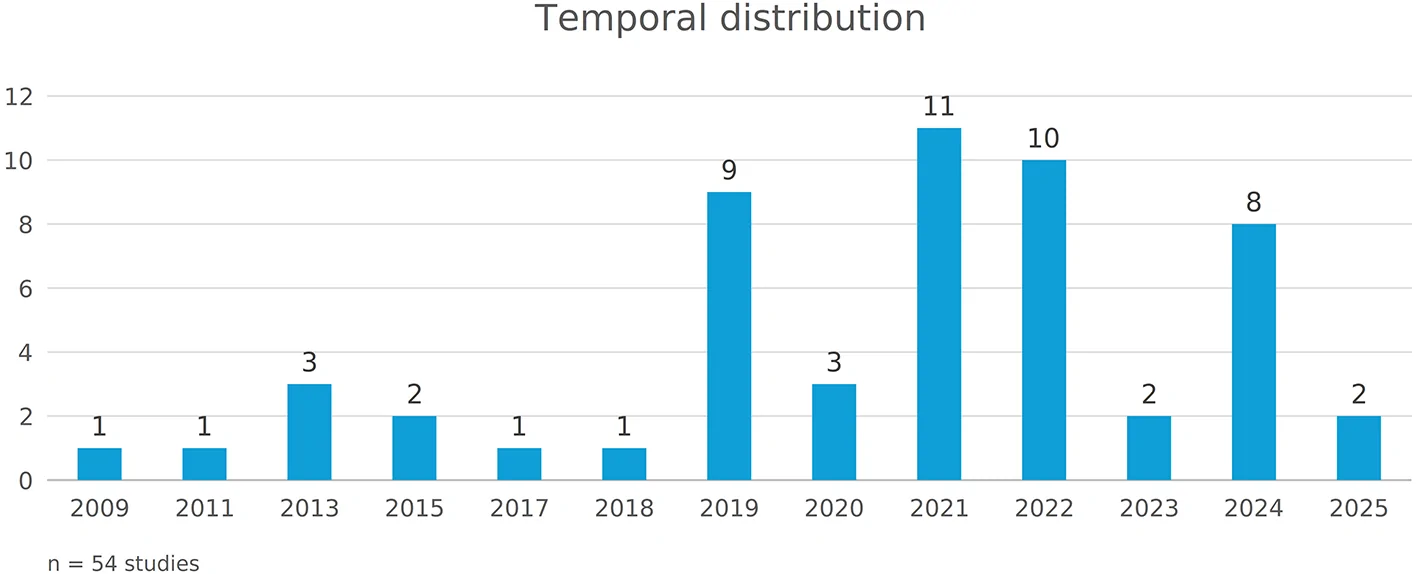
Temporal distribution.

Of the 54 selected studies, 63.0% (*n* = 34) were published between 2020 and 2024, with annual output rising notably from 2019 onward; two appeared in 2025 and the remaining 18 span 2009–2019. This recent concentration evidences a sustained increase in academic interest surrounding neuromarketing and digital marketing applied to the video game sector, particularly following the COVID-19 pandemic. This growth coincides with accelerated digitalization, the expansion of gaming as a social phenomenon, and the intensive use of digital platforms. The distribution over time is illustrated in Figure 2.

#### 2.2.2 Typology of the studies

**Figure 3:**
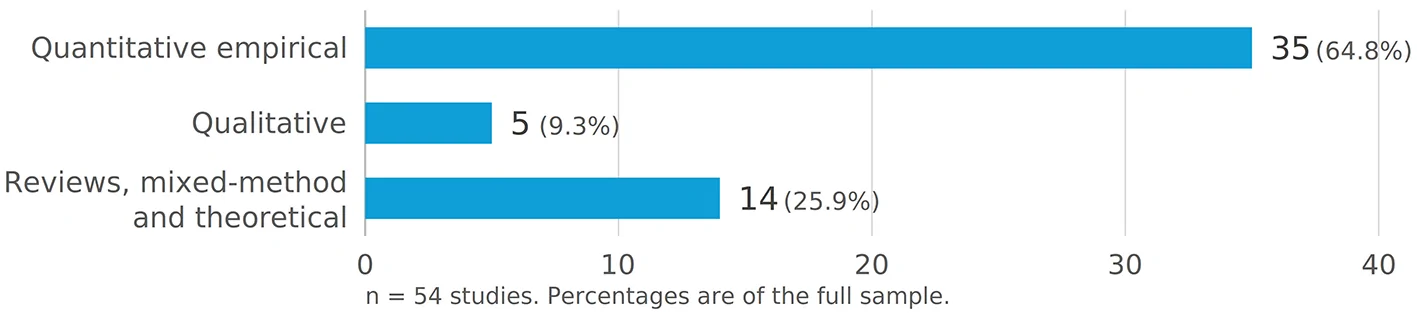
Typology of the studies.

Regarding the methodological nature of the 54 included works:

- 64.8% (35) correspond to quantitative empirical studies, including surveys, laboratory and field experiments, physiological measurement and descriptive designs.
- 9.3% (5) are qualitative studies, based on content analysis, gamer interviews, or netnography within virtual communities.
- The remaining 25.9% (14) comprise previous systematic reviews, mixed-method studies, and theoretical articles establishing conceptual frameworks on neuromarketing and digital retention. This breakdown is summarized in Figure 3.

#### 2.2.3 Disciplinary areas

The studies are distributed across three major thematic areas:

- Digital Marketing and Communication (*n* = 25; 46%).
- Cognitive Neuroscience and Consumer Psychology (*n* = 16; 30%).
- Video Game Studies and Interactive Technologies (*n* = 13; 24%).

**Figure 4:**
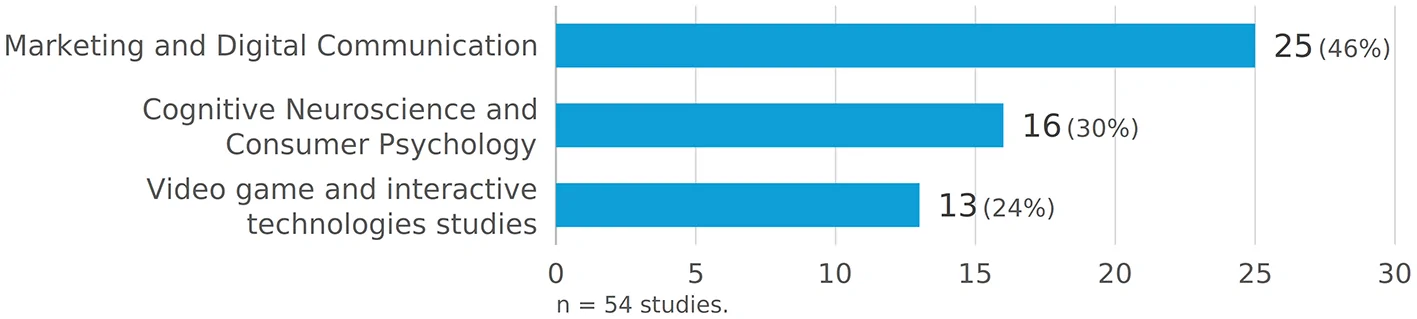
Disciplinary areas.

This distribution reflects the interdisciplinary nature of the phenomenon studied, as well as the necessity of integrating theoretical and methodological perspectives to address the complexity of gamer consumer behavior. These disciplinary areas are detailed in Figure 4.

#### 2.2.4 Geographical location

**Figure 5:**
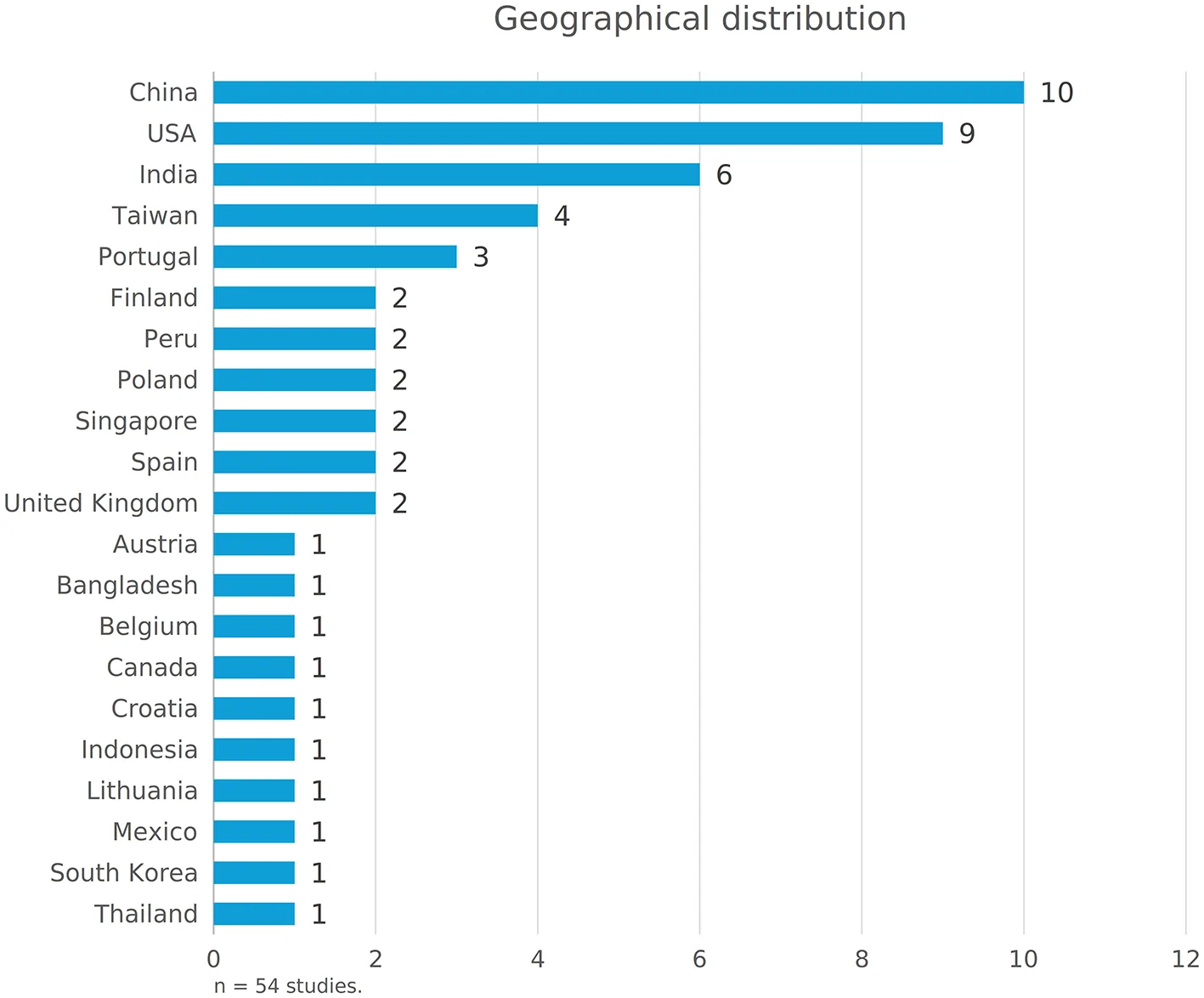
Geographical distribution.

Geographically, the corpus is internationally distributed but concentrated in two regions. The largest single national contributors are China (*n* = 10) and the United States (*n* = 9), followed by India (*n* = 6) and Taiwan (*n* = 4). Grouped by region, Europe (United Kingdom, Portugal, Spain, Finland, Poland, Belgium, Austria, Croatia, and Lithuania) and East Asia (China, Taiwan, and South Korea) account for the largest shares, followed by North America (United States and Canada), South Asia (India and Bangladesh), Southeast Asia (Singapore, Indonesia, and Thailand), and Latin America (Peru and Mexico). The Middle East and Africa are not represented. The full breakdown by country is shown in Figure 5.

Methodological quality and risk of bias were assessed for all 54 included studies as a single appraisal rather than as two independent judgements. Because a study judged to be at low risk of bias is by definition of higher methodological quality, the two vocabularies are reciprocal throughout: low risk corresponds to high quality, moderate risk to moderate quality, and high risk to low quality. Study-level ratings are reported in Supplementary Table 2, and Table 3 aggregates them by typology. Instruments were selected by design. Systematic reviews were appraised with AMSTAR 2, qualitative studies with the Joanna Briggs Institute checklist for qualitative research, and mixed-method and theoretical papers with the Critical Appraisal Skills Programme tool. For the quantitative studies, which span surveys, laboratory experiments, field experiments, physiological measurement and descriptive designs, no single published instrument covers the range of designs represented. These studies were therefore appraised against three criteria applied consistently across designs: clarity of sampling and eligibility, transparency of measurement and analytic procedure, and sufficiency of reporting for independent replication. Ratings were assigned as follows. A study was rated low risk of bias, equivalent to high quality, where all applicable criteria were met without material reservation, typically a controlled or clearly specified design with a defined sample and a replicable procedure. A study was rated moderate where the majority of criteria were met but identifiable weaknesses remained, such as a small or non-probability sample, a single-site or exploratory design, or incomplete reporting of the analysis. A study was rated high risk of bias, equivalent to low quality, where sampling, procedure or analysis could not be reconstructed from the report, or where no explicit methodological framework was given. Each study was appraised independently by two reviewers and disagreements were resolved by discussion. We acknowledge that applying common criteria to the quantitative studies is less standardized than a design-specific published checklist would be, and we identify this as a limitation in Section 4.10.

**Table 3:** Grouped quality assessment.

Study type	No. of studies	Quality rating distribution	Main limitations	Impact on conclusions
Quantitative	35 (64.8%)	High: 27 (77%) Moderate: 1 (3%) Low: 7 (20%)	Small samples in some laboratory studies; limited ecological validity; over-reliance on student and convenience samples	Carries most of the high-quality evidence, and most conclusions on attention and engagement rest on this group
Qualitative	5 (9.3%)	High: 0 (0%) Moderate: 4 (80%) Low: 1 (20%)	Lack of triangulation; subjective interpretation; small purposive samples	Adds contextual depth but supports no causal claim; conclusions drawn from this group are treated as provisional
Systematic reviews/theoretical/mixed	14 (25.9%)	High: 1 (7%) Moderate: 4 (29%) Low: 9 (64%)	Limited empirical validation; heterogeneous inclusion criteria; constituent studies not independently appraised	Useful for framing and theory development; not used as evidence of effect
Total	54 (100%)	High: 28 (51.9%) Moderate: 9 (16.7%) Low: 17 (31.5%) Note: percentages do not sum to 100 owing to rounding.		

Each study was classified into one mutually exclusive primary methodological category: experimental, quantitative (non-experimental), qualitative, mixed-methods, or theoretical/conceptual. Classification was based on the dominant research design described in the methodology section of each article to ensure internal consistency.

Given the heterogeneity of designs, outcomes, and measures across the corpus, meta-analytic pooling of effect sizes was neither appropriate nor feasible; we therefore followed guidance for Synthesis Without Meta-analysis (SWiM), using structured tabulation and direction-of-effect (“vote-counting”) summaries stratified by appraised quality, rather than pooled estimates. To prevent double-counting, each study was assigned to a single, mutually exclusive primary methodological category for all quantitative tallies; although a study may be discussed under more than one thematic heading, it contributes only once to the counts reported in Figures 2–5 and to the quality distribution.

## 3 Results

### 3.1 Study characteristics and methodological approaches

The final sample comprised 54 studies spanning 2009 to 2025, with output accelerating notably from 2019 onward. This temporal clustering reflects the convergence of two independent forces: the post-COVID-19 intensification of digital gaming behavior, and the maturation of social media as both a marketing channel and a research context. Methodologically, the studies were heterogeneous. Experimental designs (both laboratory-controlled and field-based) accounted for the largest share, supplemented by large-scale surveys, mixed-method protocols, and theoretical or integrative reviews. Sample sizes spanned from focused qualitative inquiries involving 14 to 24 participants to large-scale experiments and surveys exceeding 1,000 respondents, providing breadth alongside depth.

To avoid conflating evidence of differing ecological proximity to gaming, we distinguish four classes of study within the corpus: (i) general advertising and retail neuromarketing studies conducted outside gaming, included primarily as methodological models; (ii) game-related advertising studies examining in-game advertising, advergames, or game-adjacent promotional content; (iii) actual gameplay studies measuring responses during play; and (iv) field or longitudinal studies linking measured responses to subsequent player behavior. The corpus is weighted toward classes (i) and (ii); studies of class (iii) are fewer, and class (iv), the only design capable of validating retention claims over weeks or months, is rare. Accordingly, short-horizon physiological or self-report responses reported here should not be read as demonstrated predictors of long-term retention in the absence of longitudinal confirmation.

Across these works, two complementary methodological streams emerged: (a) behavioral and self-report approaches quantifying attitudes, intentions, loyalty, and engagement; and (b) physiological and neuromarketing approaches using tools such as EEG, eye-tracking, and biometric measures to capture implicit processes. Neuromarketing tools were synthesized in systematic reviews that highlighted EEG, eye tracking, and facial recognition as dominant technologies used to understand consumer decision-making and emotional responses. Together, these methods enabled the evaluation of both conscious and non-conscious mechanisms through which digital marketing strategies and neuromarketing influence video game consumer behavior and retention.

### 3.2 Neuromarketing tools and implicit responses relevant to gaming

Pioneering neurophysiological work demonstrated that EEG and galvanic skin response can detect subtle emotional activation and attention to advertising stimuli beyond self-report, providing a robust basis for measuring emotional memory and ad effectiveness (Ohme et al., 2009). Subsequent systematic reviews consolidated the prominence of EEG, eye-tracking, and facial recognition in neuromarketing, emphasizing their potential to reveal unconscious drivers of consumer response while calling for clear ethical and methodological standards (Bhardwaj et al., 2024; Rawnaque et al., 2020). In an immersive virtual retail environment (not gaming), eye-tracking combined with pupillometry showed that visual attention and arousal varied systematically with the spatial arrangement of store elements, although attention and arousal were correlated for only one area of interest in one of the two stimuli, and purchase outcomes were not measured (Kim and Lee, 2021). The transferability of such retail findings to interactive game environments therefore remains to be tested directly.

It is important to distinguish what each technique measures directly from what is subsequently inferred. Electroencephalography records scalp electrical activity, from which indices of attention, cognitive load, and approach/withdrawal-related affect are inferred; it has limited spatial resolution and is sensitive to movement and ocular artifacts. Eye-tracking records overt gaze and fixation, indexing where visual attention is directed, but does not by itself indicate preference, comprehension, or positive valence. Pupillometry indexes arousal and cognitive effort but is strongly confounded by luminance and contrast. Electrodermal (galvanic skin) activity indexes sympathetic arousal intensity but not valence, and so cannot distinguish positive from negative affect. Facial coding infers discrete emotions or valence from facial-muscle configurations, but is affected by display rules, cultural variation, and the imperfect correspondence between expressed and felt emotion. Convergent, multi-method evidence is therefore more informative than any single index, and inferences about complex states such as “engagement” or “preference” should be drawn cautiously.

These neuromarketing tools offer particular relevance to the video game sector, where highly immersive and visually complex environments shape player engagement and retention. By capturing continuous, implicit responses during exposure to game interfaces, in-game advertising, and game-related content, neuromarketing methods complement traditional surveys and experiments and enable more precise modeling of drivers of continued usage and loyalty (Bhardwaj et al., 2024; Rawnaque et al., 2020). The evidence therefore supports the use of combined physiological and behavioral metrics for comprehensive and empirically validated evaluation of digital marketing strategies in gaming contexts.

Behl et al. (2024) approach the same convergence from the practitioner side, surveying 267 digital marketing strategists at retail firms and modeling, through structural equation modeling, the relationships they perceive between cognitive neuroscience, gameful experience, intrinsic motivation and sustained behavior change. Their contribution is a Self-Determination Theory framework for combining gamification with neuromarketing, together with evidence that practitioners adopt these tools in the belief that they yield more precise results and reduce marketing costs. Because the respondents are marketing professionals rather than consumers or players, and the setting is retail rather than gaming, the study speaks to how these techniques are adopted rather than to their effects on player engagement. Similarly, Gupta et al. (2025) propose a stage-specific framework mapping neuromarketing tools onto distinct phases of the consumer journey, from initial awareness to post-purchase loyalty maintenance, an architecture directly applicable to the retention-focused scope of the present review.

### 3.3 Digital marketing strategies, engagement, and retention

A large group of studies examined how digital marketing activities on social media and platform-based ecosystems shape engagement, brand attitudes, and downstream behavioral intentions that are directly tied to retention. In social commerce settings, customer identification with online communities increased platform usage, purchase behaviors, and loyalty, underscoring the role of community-based identification in sustained engagement (Shin, 2013; Wang et al., 2015). Similarly, active participation in social media-based brand communities was shown to foster brand love and stronger brand relationships, which are key antecedents of long-term retention (Lee and Hsieh, 2022; Wong, 2021).

Several studies demonstrated that social media marketing activities (e.g., informative, interactive, and experiential content) enhance consumer-based brand equity, brand love, and positive customer responses, thereby increasing the likelihood of continued usage (Chen and Qasim, 2021; Hofmann et al., 2021; Naeem, 2019; Pehlivan et al., 2011; Upadhyay et al., 2022). Viral marketing and short video ads were found to boost brand recognition, preference, and sales when content was engaging and strategically distributed, suggesting that dynamic formats can reinforce repeated interactions and usage intentions (Ge et al., 2021; Puriwat and Tripopsakul, 2021; Souki et al., 2022). Studies also showed that live-streamed social commerce and real-time interactions in live streams positively influenced decision-making and purchasing, highlighting immediacy and social presence as drivers of ongoing engagement (Fletcher and Gbadamosi, 2022).

### 3.4 Influencers, content quality, and trust transfer

Influencer marketing emerged as a central mechanism through which digital strategies shape trust, engagement, and purchase intentions, thereby affecting retention. Quantitative studies revealed that endorsements by credible influencers increased app adoption and brand-related behavioral intentions through trust transfer, where trust in the influencer generalized to the endorsed product (Hu et al., 2019; Torres et al., 2019). A dedicated influencer index integrated metrics from Facebook, Twitter, and Instagram to predict influencer impact, showing that reach, interaction, and perceived authenticity are key to forecasting engagement and potential retention (Arora et al., 2019).

In adolescent and youth segments, exposure to influencers whose content and credibility were perceived as high strengthened parasocial relationships and materialistic values, which, in turn, increased purchase intentions; however, parental mediation mitigated these effects, indicating boundary conditions for long-term influence (Lou and Kim, 2019). Research on YouTube content creators showed that YouTubers' attitudes and content strongly shaped the impact of commercial advertising, revealing that creator-audience relationships critically influence how advertising is processed and whether viewers remain engaged (Larios-Gómez and Fischer, 2024). Studies on brand anthropomorphism demonstrated that human-like brand communication on social networking sites could foster consumer brand evangelism, especially for prevention-focused individuals, further supporting retention through strong emotional bonds (Zhang and Choi, 2023).

### 3.5 Visual and narrative design: congruence, attention, and memory

Visual quality and congruence between marketing stimuli and contextual content were consistently associated with enhanced engagement and memory, which are precursors to retention. Experiments on Instagram marketing showed that high image quality and positive social cues improved perceived product quality and purchase intention, providing evidence of visual design effects on behavioral outcomes (Teo et al., 2019). Content analyses and experiments indicated that visually congruent influencer posts and brand elements increased brand engagement and interaction over time (Argyris et al., 2020; Cuevas-Molano et al., 2021; Karpenka et al., 2021; Zhao et al., 2022).

In game-related settings, in-game advertising and advergames were compared in terms of persuasive effectiveness, with advergames generating stronger engagement but in-game placements performing better when congruent with the game narrative (Ghosh et al., 2022b). A complementary experiment contrasted logos and brand names embedded in games, reporting that pictorial logos attracted more attention and fostered spontaneous recall, whereas textual brand names supported recognition, both of which are important to cumulative brand memory and potential loyalty (Ghosh et al., 2022a). In Instagram Stories with game ads, greater game involvement increased visual attention to embedded advertisements, suggesting that involvement moderates attention to cross-platform promotional content that may sustain interest in games (Huang and Gong, 2024). Related work in game-specific settings has examined the interaction between branded content and play (Elias et al., 2015), player perception of a specific in-game placement (Rodríguez-Ramírez et al., 2021), and the curiosity and social-comparison pathways through which game advertising shapes willingness to play (Zetian et al., 2024).

A more recent systematic review by Zagni et al. (2025) extends these findings by mapping the convergences and divergences between advergames and in-game advertising across a broader body of evidence. Their analysis highlights cognitive load and perceived intrusiveness as the two most consistent moderators of in-game advertising effectiveness (variables that were measured inconsistently across the earlier studies in this review's sample) and calls for standardized operationalization of these constructs in future experimental work.

Several findings in the corpus diverge or are moderated in ways that caution against uniform conclusions. In-game advertising and advergames differ in persuasive effect according to narrative congruence, with advergames generating stronger engagement but in-game placements outperforming when congruent (Ghosh et al., 2022b); pictorial and textual brand elements trade off attention and spontaneous recall against recognition (Ghosh et al., 2022a). Influencer effects on adolescents were attenuated by parental mediation, indicating boundary conditions rather than uniform impact (Lou and Kim, 2019). Moderators such as cognitive load and perceived intrusiveness were operationalized inconsistently across studies, limiting comparability (Zagni et al., 2025). These discrepancies, together with variation in measurement and design quality, argue against pooling and in favor of a structured, quality-weighted narrative synthesis.

### 3.6 Game experience, community, and retention-specific outcomes

The reviewed work pointed to specific game- and community-related mechanisms that directly support video game retention. Esports spectating was positively associated with game consumption and purchasing intentions: viewers who identified more strongly with esports players and teams exhibited greater gameplay and in-game spending, linking spectator engagement with ongoing usage (Macey et al., 2022). New game design elements, creatability, achievability, and immersibility, were found to increase online game usage, player satisfaction, and continuance intention in longitudinal research, indicating that design features themselves function as retention levers (Teng et al., 2024).

Studies on fanwork creation and transmedia storytelling in mobile games showed that moving consumers to prosumers through co-creation and narrative extension strengthened emotional bonds and loyalty, promoting long-term engagement with the game world (Cui et al., 2022; Yucra-Quispe et al., 2022). Peer-initiated brand communities and social media brand communities were linked to brand love, attachment, and loyalty, indicating that community engagement around games and game-related brands fosters relationship quality essential for retention (Lee and Hsieh, 2022; Wong, 2021). In addition, studies on brand-related values and video sharing revealed that value congruence and entertainment-oriented sharing behaviors enhanced brand equity and attachment, indirectly supporting ongoing engagement (Nikolinakou and Phua, 2019; Souki et al., 2022).

Some works explicitly addressed game-as-a-service dynamics and service withdrawal, noting that uncertainty and lack of transparency around service termination can erode trust and weaken long-term loyalty to digital games and platforms (Dubois and Chalk, 2024). Research on perceived deception in online games found that deceptive elements in game design negatively affected motivation and choice, implying that ethical and transparent mechanics are crucial for sustaining players over time (Zhao and Thadani, 2021). Interview-based research on digital services further revealed that everyday digital services (including games) can simultaneously support and undermine wellbeing, pointing to a dual role of digital entertainment in perceived quality of life and potentially in retention (Kemppainen and Paananen, 2024).

Extending these findings, Ascarza et al. (2025) provide rare field-experimental evidence from a freemium mobile game, randomizing personalized difficulty reduction among 329,999 low-activity players over 50 days with 30 days of follow-up. Easing difficulty reduced immediate in-round purchasing, since a less demanding game substitutes for buying advantages, but it increased engagement and retention and, through them, total spending, yielding a net gain of roughly seven to eight cents per user over 30 days. The authors therefore report a synergy between retention and monetization where a substitution is usually assumed. Critically, their results suggest that the commonly assumed tension between free-to-play access and paid conversion can be partially resolved through adaptive design, rather than through marketing persuasion alone. This shifts the analytical emphasis from external campaign design to embedded product mechanics as a form of marketing intervention, a perspective underrepresented in the reviewed literature.

### 3.7 Trust, CSR, and ethical dimensions

Trust emerged as a cross-cutting factor linking digital marketing to long-term behavioral outcomes. Social media advertising research showed that alignment between users' motivations and ad congruency fostered perceptions of trust in social media advertising, thereby supporting positive responses and repeat interactions (Carlson et al., 2021; Lee et al., 2019). Surveys on CSR-related activities in social media reported that corporate social responsibility initiatives communicated online improved brand attitude, electronic word-of-mouth, and purchase intention, suggesting that ethical positioning enhances relational outcomes that favor retention (Chu and Chen, 2019).

Several studies further emphasized the role of sponsorship disclosure, user values, and parental or regulatory mediation in shaping how persuasive content is processed over time. For instance, disclosure of sponsorship affected Generation Z's purchase intentions, indicating that transparent labeling influences the perceived legitimacy of marketing messages (Sesar et al., 2023). Meta-analytic work on advertising value concluded that credibility and entertainment are core drivers of perceived advertising value, corroborating that enjoyable and trustworthy communication underpins favorable attitudes and behavioral intentions across contexts (Ye et al., 2025). Finally, policy and review work on youth media use underscored the potential health and developmental risks of intensive exposure to digital media and persuasive content, reinforcing the need for ethical neuromarketing and responsible digital marketing practices in youth-oriented video game environments (Strasburger et al., 2013; Hinsch and Sheldon, 2013).

### 3.8 Quality assessment and impact on conclusions

Overall, 51.9% of the studies were rated high quality, that is at low risk of bias, 16.7% moderate, and 31.5% low. Quantitative studies (*n* = 35) demonstrated strong internal validity through controlled designs and the use of neuromarketing tools (EEG, eye-tracking), and account for most of the high-quality evidence, though ecological validity was often limited due to laboratory environments. Qualitative studies (*n* = 5) offered rich insights but were rated moderate or low throughout, since they frequently lacked triangulation and standardized coding procedures. Systematic reviews, theoretical works and mixed (*n* = 14) offered valuable conceptual synthesis but were predominantly rated low, due to their restricted empirical validation and heterogeneous inclusion criteria. Table 3 groups quality assessments attending to their typology.

The direction of reported associations across outcome families is summarized in Table 2. Reported associations are predominantly positive but unevenly distributed: evidence is concentrated in engagement, trust and loyalty, and purchase intention, and is comparatively sparse for memory, arousal, and retention. No general-advertising or during-gameplay study in the corpus addressed retention directly; the retention row is populated only by game-related and field or longitudinal studies, of which two (Teng et al., 2024; Ascarza et al., 2025) employed designs capable of tracking behavior over time. Mixed and negative findings cluster in the engagement and loyalty outcomes.

### 3.9 Demographics of the publications

The demographic analysis of the publications included in this systematic review reveals significant trends regarding the chronology, study typology, geographical origin of the research, publishing journals, and predominant academic disciplines.

## 4 Discussion

### 4.1 What this review adds

Beyond cataloging strategies and tools, this review makes three contributions. First, it organizes a fragmented, cross-disciplinary evidence base around retention as the focal outcome and separates measured outcomes from inferred antecedents. Second, it distinguishes static neuromarketing measurement (discrete, laboratory-based assessment of responses to fixed stimuli) from dynamic, neuromarketing-informed design (real-time, personalized adaptation of game mechanics), arguing that the latter raises distinct measurement and ethical questions (Section 4.9). Third, it specifies the evidential conditions (longitudinal, ecologically valid, gaming-specific designs) under which current associations could be upgraded to causal, retention-level claims. The subsections below develop these points rather than restating the results.

The discussion of this systematic review is organized into four emerging thematic axes: (1) the evolution and characterization of the video game sector; (2) digital marketing strategies applied to consumer retention; (3) the profile of the consumer and purchasing behavior; and (4) the application of neuromarketing and emotions in decision-making. Each axis integrates the most relevant theoretical and empirical contributions found in the selected studies, including those key references from the original document.

### 4.2 Evolution of the video game sector and market transformation

The sector's transformation into a global cultural economy is the condition that makes the retention problem acute rather than a finding of this review. Scale and diversification, already documented in the Introduction, matter here for one reason: they multiply the number of competing claims on player attention, which is precisely what makes implicit, pre-reflective measures attractive to marketers and ethically consequential for players.

Moreover, there has been a growing sophistication of the business ecosystem, which now includes developers, publishers, distributors, and platforms reorganized under subscription-based business models (e.g., Game Pass) and microtransactions. The influence of emerging markets such as India and Latin America is shifting content and design dynamics, demanding more tailored and localized experiences.

### 4.3 Digital marketing strategies and consumer retention

The evolution of Web 2.0 and the rise of social networks have made digital marketing the cornerstone of business strategy in the gaming sector. Kotler and Armstrong (2004) and Mendoza et al. (2007) highlight the transition from a unidirectional model to bidirectional communication focused on content co-creation. Social platforms allow companies to showcase brand values, build emotional narratives, and foster sustainable relationships with their consumers.

During the COVID-19 pandemic, this approach was consolidated through intensified SEO, SEM, and targeted advertising, generating real-time data on user preferences (Levitt, 1975; Malfitano Cayuela, 2007). Digital loyalty has been particularly strengthened by user-generated content, whose perceived legitimacy surpasses that of corporate advertising (Bigné and Andreu, 2004).

In parallel, branded content and the role of influencers and streamers have emerged as key catalysts for engagement. According to Keller and Kotler (2006), these opinion leaders act as credible mediators, mobilizing specific communities. The parasocial relationship, based on the perception of authenticity and closeness, has become the emotional foundation of the brand bond (García Galicia, 2010; Arora et al., 2019).

### 4.4 The new gamer consumer: roles, motivations, and decision-making

The figure of the consumer has evolved from a passive recipient to a prosumer user, actively involved in the creation and dissemination of content (Chan and Astari, 2017). This new profile seeks more personalized and relevant experiences (Abbas, 2024), which has led to massive investment in algorithmic tools for segmentation and recommendation.

Trust has become a decisive intangible asset in today's oversaturated digital environments (Saveljeva and Volkova, 2025). Various studies confirm that consumers no longer base their decisions solely on brand reputation but increasingly rely on social actors, such as influencers and reviews, to validate their choices (Torres et al., 2019). More than 70% of buyers link their purchase decision to trust factors, including “good reviews” and the brand's social reputation (Edelman, 2019), a trend amplified by the viral effect of social networks (Puriwat and Tripopsakul, 2021).

Regarding user types, Kallio et al. (2011) describe an engagement continuum that functionally groups three major profiles (casual, enthusiast, and professional) each driven by different motivations and expectations; hence the need to design differentiated content and engagement strategies.

To refine these strategies, it is crucial to measure the actual attention of each group: eye-tracking studies in virtual shopping environments has shown that dwell time, fixation count, and pupil diameter differ significantly between male and female participants for specific store elements, indicating that attentional and arousal responses are not uniform across a target audience (Kim and Lee, 2021). Whether comparable differences hold across other segmentation criteria, or across player types in gaming, has not yet been tested.

### 4.5 Emotions, neuroscience, and consumer retention

The role of emotions in decision-making has been extensively documented in the context of video game marketing. Toh (2021) and Martínez-Maldonado (2025) provide contemporary insights into how decision-making processes operate within gaming environments, highlighting the influence of cognitive and emotional mechanisms on player engagement and in-game choices. Prehn-Kristensen et al. (2009) demonstrated the relationship between emotional stimuli and information retention, which directly translates into engagement and brand loyalty. Ohme et al. (2009) argue that intense emotions, such as euphoria or empathy toward characters, have a direct impact on player satisfaction. From a visual perspective, Rizun and Kucharska (2018) and Bonetti et al. (2016) highlight that visual stimuli (colors and graphics) significantly influence the aesthetic and functional perception of the game. This perception translates into recall, a critical variable in retention, as supported by Braidot (2006) and Malfitano Cayuela (2007).

Neuromarketing has enabled deeper exploration of these processes through techniques such as eye tracking, EEG, face ID, or fMRI (Bonadeo, 2005; Rawnaque et al., 2020; Bhardwaj et al., 2024). The combination of these technologies with artificial intelligence has enhanced brands' ability to interpret emotions and behaviors in real time (Liu-Thompkins et al., 2022).

Cases such as *The Last of Us Part II* and the remakes of *Final Fantasy VII, Resident Evil 2*, or *The Legend of Zelda: Link's Awakening* demonstrate the power of emotional storytelling, where memory and emotion are integrated into brand strategy (Ohme et al., 2009; Saura et al., 2020). However, ethical risks regarding the subconscious manipulation of consumers have also been noted (Saura et al., 2020), opening debates about transparency and corporate responsibility.

The impact of study quality on conclusions was evident: high-quality studies consistently provided robust evidence on emotional engagement and retention mechanisms, whereas low-quality studies tended to generalize findings without sufficient empirical grounding. This variability underscores the importance of cautious interpretation when applying neuromarketing insights to strategic decision-making in the video game sector.

The findings obtained from the systematic analysis of the literature confirm that the video game sector has transcended its purely recreational dimension to become an ecosystem of high cultural, economic, and emotional value. In this context, digital marketing and neuromarketing emerge as key tools to understand and shape consumption dynamics in a highly competitive, fragmented, and emotionally charged environment.

### 4.6 Critical appraisal of included studies

The systematic review included a total of 54 studies, whose methodological quality and thematic relevance were analyzed in terms of design, rigor, and applicability to the objectives of this review.

Most studies correspond to quantitative designs (64.8%), including controlled experiments, structured surveys, and studies using techniques such as eye-tracking. These provide good accuracy for measuring variables like attention, engagement, and purchase intention; however, several present limitations such as small sample sizes, highly controlled laboratory environments, or an overreliance on student populations, which restricts the generalizability of findings.

Qualitative studies account for 9.3%, focusing on content analysis, interviews, or netnography. While offering contextual depth, some lack methodological triangulation or inter-researcher validation, reducing reliability, and none was rated high quality. The remaining 25.9% are systematic reviews, mixed-method and theoretical studies, which provide valuable conceptual contributions but limited empirical validation, and were predominantly rated low.

In some cases, research design descriptions were brief or not fully standardized, requiring interpretative classification based on methodological details provided. This ambiguity often stems from vague descriptions or exploratory approaches without a systematic framework.

In terms of thematic relevance, studies were carefully refined to ensure alignment with the review's core focus: the intersection of neuromarketing, digital marketing, and consumer behavior in video game contexts. Nevertheless, common limitations include the lack of longitudinal studies, heavy geographic concentration in East Asia, Western Europe and North America, and the underrepresentation of emerging markets.

Despite these issues, several studies stand out for the rigor of their designs, including a randomized field experiment with behavioral outcome measures (Ascarza et al., 2025) and controlled psychophysiological work in immersive commercial environments (Kim and Lee, 2021), the latter conducted outside gaming and without behavioral outcome measures.

**Figure 6:**
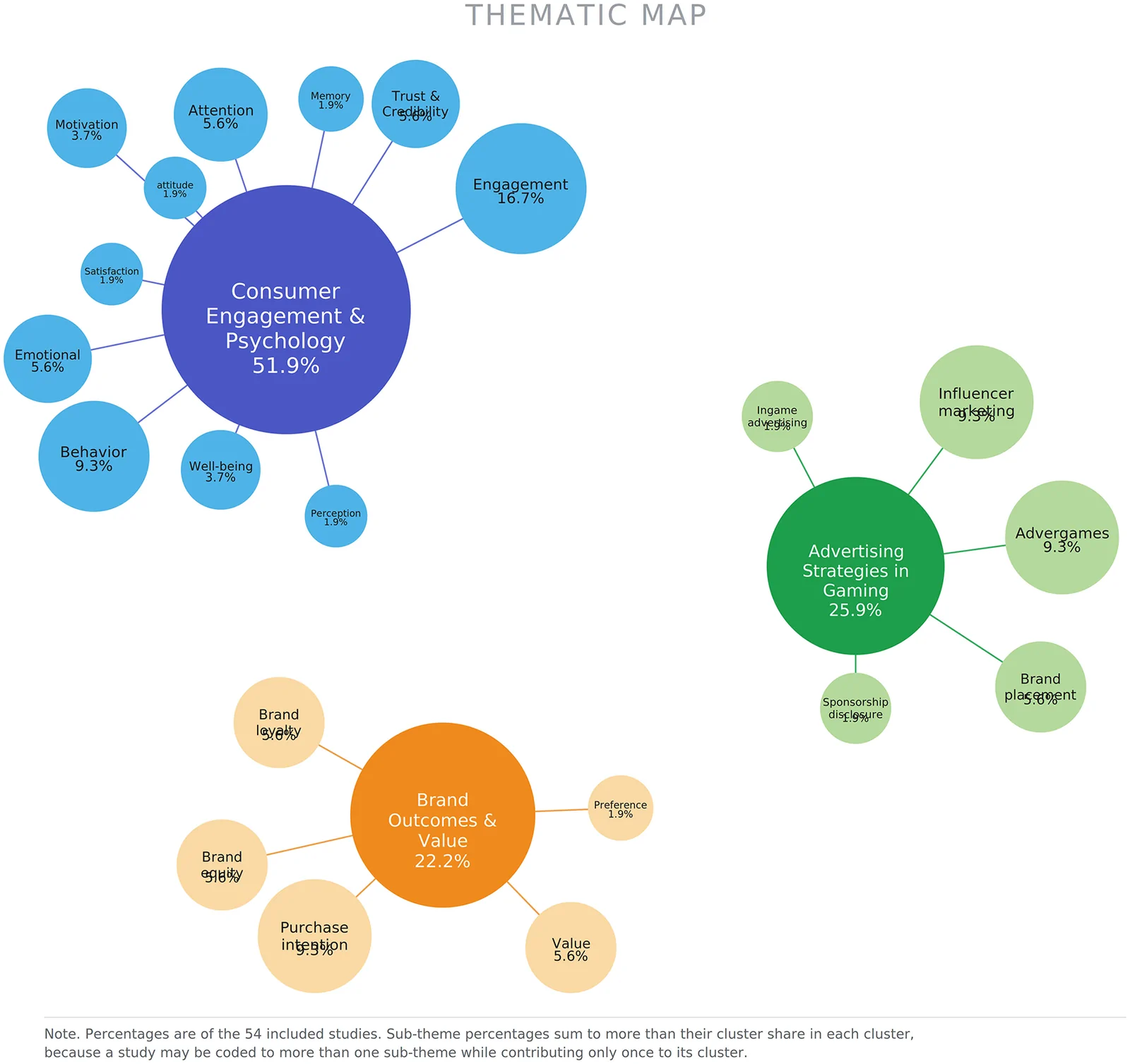
Thematic map.

Figure 6 represents a thematic map for the 54 reviewed studies. It visually synthesizes the conceptual structure that emerged from this systematic review. Three overarching thematic domains were identified and are reflected in the diagram: Consumer Engagement & Psychology, Advertising Strategies in Gaming, and Brand Outcomes & Value. Each of these clusters encapsulates key subdimensions frequently addressed in the literature, and their relative size in the map corresponds to the number of studies discussing them.

The largest cluster, Consumer Engagement & Psychology, represents 51.9% of the included studies (*n* = 28) and encompasses constructs such as emotional engagement, attention and memory processes, perceived quality, trust, and motivational or behavioral responses. These aspects reflect how users cognitively and emotionally react to marketing strategies embedded in gaming environments.

The second cluster, Brand Outcomes & Value, accounts for 22.2% of the studies (*n* = 12) and includes outcomes such as brand loyalty, purchase intention, brand equity, social media influence, and community engagement. These variables often serve as indicators of long-term consumer response and are commonly used to evaluate the impact of marketing interventions on brand positioning.

Finally, the cluster labeled Advertising Strategies in Gaming (25.9%; *n* = 14) groups together studies that examine the various techniques used to deliver marketing messages in games. This includes in-game advertising, influencer campaigns, advergames, brand placement, and sponsorship disclosures, each aiming to integrate commercial content within digital gaming ecosystems in ways that enhance persuasion and exposure.

The thematic map highlights the interconnections between these three domains. Advertising strategies often serve as stimuli that activate psychological mechanisms, which in turn influence brand-related outcomes. The visual proportion of each thematic node offers a clear representation of the prevalence of these topics across the body of research analyzed.

### 4.7 Convergence between digital marketing and consumer neuroscience

One of the most relevant findings of this review is the progressive convergence between digital marketing strategies and neuromarketing principles. Although these disciplines have traditionally developed separately, the reviewed studies show a clear trend toward their integration to design immersive and emotionally meaningful experiences. Emotions, visual attention, and memory emerge as central variables in this process (Rizun and Kucharska, 2018).

This integration translates into advertising campaigns that appeal to authenticity (Bigné and Andreu, 2004), user-generated content, and parasocial relationships with streamers and influencers (García Galicia, 2010; Arora et al., 2019). Brands no longer operate merely as message broadcasters but as architects of emotional bonds, aligning with the perspectives advocated by Kotler and Armstrong (2004) and Levitt (1975) on the evolution of relational marketing.

### 4.8 The consumer as an active and emotional agent

The study of the gamer consumer reveals an increasingly complex figure, autonomous and emotionally involved. This consumer, according to Chan and Astari (2017), actively participates in building brand reputation through reviews, gameplays, and recommendations, reshaping the notion of authority in purchasing decisions. Trust is no longer constructed exclusively by the company but by the user community, whose validation is perceived as more legitimate.

Adding to this is the phenomenon of hyper-segmentation, where companies tailor their strategies to the player profiles described above (Kallio et al., 2011). Each segment responds to different motivations, so retention must be addressed through multiple pathways rather than a single lever.

### 4.9 Emotional retention and ethical boundaries of neuromarketing

One of the most striking findings is the use of neuromarketing as a mechanism for emotional retention. The literature shows that consumers remember and value more highly those experiences that generate a strong positive emotional charge (Ohme et al., 2009; Braidot, 2006). This is reflected in practices such as nostalgic storytelling, eye tracking techniques, and the design of highly personalized visual experiences.

However, this approach raises important ethical tensions, especially when strategies affect decision-making subconsciously. Saura et al. (2020) warn about the fine line between influencing and manipulating, calling for regulatory frameworks to govern the use of neurocognitive technologies for commercial purposes. In this regard, companies must balance performance optimization with the preservation of consumer autonomy.

The deployment of dynamic personalization systems, such as the personalized difficulty adjustment documented by Ascarza et al. (2025), raises a distinct and more operationally specific concern than traditional neuromarketing. Such systems adapt game mechanics to individual behavioral profiles without explicit disclosure to the player, and the evidence indicates that they can alter spending through changes in engagement and retention rather than through direct persuasion, a pathway the player is unlikely to perceive. Behl et al. (2024) show that practitioners are actively interested in combining gamification with neuromarketing for precisely such purposes, which suggests that this form of intervention is likely to become more common. Unlike a fixed advertisement measured in a laboratory, these interventions are continuous, personalized, and embedded within an experience the player has actively chosen. The regulatory frameworks cited in earlier literature (Murphy et al., 2008; Fisher et al., 2010) were not designed with this architecture in mind. Future regulatory and ethical scholarship will need to distinguish between static neuromarketing measurement and dynamic neuromarketing-informed design, as the latter represents a qualitatively different form of commercial influence.

### 4.10 Theoretical and methodological gaps

Despite the growing body of literature examined here, three structural gaps emerge with consistency. The first concerns temporal validity: the overwhelming majority of studies employ cross-sectional or short-horizon experimental designs, meaning that claims about emotional retention or brand loyalty are based on immediate post-exposure measures rather than sustained behavioral change. The need for longitudinal designs tracking real player behavior over months or seasons remains unmet, and this limits the practical value of neuromarketing findings for game developers working with live-service titles.

The second gap concerns ecological validity. Laboratory applications of EEG and eye-tracking, while methodologically rigorous, are conducted under conditions that diverge substantially from actual gaming sessions (i.e., interrupted, multi-screen, socially embedded experiences). The recent shift toward web-platform neuromarketing reviewed by Cenizo (2025) suggests that ecological deployment of these tools is technically feasible, but gaming-specific deployments in naturalistic settings remain rare. Third, and most persistently, the geographic concentration of studies in North America, Western Europe, and East Asia means that the emotional and behavioral dynamics documented here may not generalize to the fastest-growing gaming markets globally, such as LATAM and MENA, where cultural attitudes toward advertising, community, and brand trust diverge in ways the existing literature has not examined.

Two further limitations concern the review process itself: search coverage and appraisal:

The review draws on three bibliographic databases. Because Scopus and the Web of Science Core Collection index the great majority of journal content from publishers whose own platforms were not searched separately, we judge the resulting coverage loss to be limited; one included study, for instance, appeared in IEEE Access and was retrieved through Web of Science. Platform-specific searching, particularly of conference proceedings, could nonetheless have identified further records.

The heterogeneity of designs in the corpus meant that no single published instrument could be applied to the quantitative studies, which were instead assessed against common criteria of sampling clarity, procedural transparency and replicability. While these criteria were applied consistently and independently by two reviewers, they are less standardized than a design-specific checklist, and quality ratings for this group should be read with that in mind.

### 4.11 Practical and academic relevance

From a practical standpoint, the findings of this review provide strategic insights for video game designers, marketing specialists, and technology companies. Emotional personalization, influencer relationships, and a neurocognitive understanding of the player are the mechanisms most frequently associated with engagement and with the antecedents of retention. Their effect on retention itself is not established by the present evidence base, in which only two studies tracked player behavior over time.

From an academic perspective, this review offers an integrative theoretical framework that can guide future empirical research on video game consumption from an emotional and cognitive viewpoint, incorporating behavioral data, physiological responses, and interactive experiences.

### 4.12 Ethical considerations in neuromarketing applied to the video game sector

The use of neuromarketing in digital environments raises significant ethical concerns, particularly when strategies influence decision-making in a subconscious or emotionally manipulative manner. Several theoretical frameworks address these dilemmas from the perspectives of neuroethics and consumer psychology. For instance, Murphy et al. (2008) warn of the risk of “neuroexploitation” when neuroscientific tools are applied without informed consent or without respecting consumer autonomy. Similarly, Fisher et al. (2010) emphasize the need for transparency and regulation regarding the use of technologies such as EEG and eye-tracking in commercial contexts.

Within this review, some of the included studies (e.g., Rawnaque et al., 2020) employ techniques like eye-tracking and emotional response analysis to measure the effectiveness of advertisements in video games. While these methods offer high precision, they can also be used to design highly persuasive experiences without users being fully aware of the mechanisms involved. This issue becomes particularly critical in the video game sector, where the high level of immersion and the relatively young profile of many users increase vulnerability to such influences.

Therefore, it is essential to integrate ethical principles into the design of neuromarketing strategies, especially regarding sensitive populations (such as minors), the responsible use of biometric data, and the implementation of informed consent frameworks adapted to the digital environment. Including these safeguards not only strengthens the legitimacy of marketing practices but also contributes to a more sustainable and user-centered approach.

## 5 Conclusions

Four conclusions follow from this analysis. Digital marketing has fundamentally restructured how gaming companies and consumers relate to one another. The shift from broadcast advertising to participatory, community-embedded communication means that brand relationships now depend on user co-creation and peer validation as much as on corporate messaging, a development that both expands and complicates the marketer's toolkit.

Neuromarketing offers differential value in this ecosystem to the extent that it captures what survey instruments cannot: the implicit, pre-reflective responses associated with attention allocation, memory encoding, and affective response during gameplay. The reviewed evidence suggests that techniques such as EEG, eye-tracking, and facial coding, when applied rigorously, can identify engagement drivers invisible to self-report methods.

The contemporary gamer is not a passive recipient of designed experiences but an active agent whose decisions are shaped by social networks, parasocial relationships, community norms, and platform architecture simultaneously. Strategies that treat retention as a function of a single variable (whether engagement metrics, incentive design, or emotional narrative) will underperform relative to those that model this multidimensional reality.

Finally, the methodological infrastructure of this field remains uneven. High-quality experimental work provides robust evidence on specific mechanisms, but the absence of longitudinal designs, the limited ecological validity of laboratory neuromarketing, and the geographic concentration of the evidence base collectively restrict the generalizability of current findings. Addressing these limitations is not merely an academic concern; it is a precondition for the responsible application of neurocognitive tools in commercial gaming contexts.

Taken together, the evidence indicates that neuromarketing measures yield reliable insight into immediate attention and engagement, but that their capacity to enhance long-term player retention is not established by the studies reviewed here. Direct evidence on retention comes from two studies with longitudinal or field designs, against a corpus weighted toward short-horizon measurement and, in a substantial minority of cases, toward non-gaming populations.

### 5.1 Practical implications

The findings of this review have important implications for the video game industry:

- Brands should design strategies based on genuine emotional bonds, appealing to values such as authenticity, empathy, and nostalgia.
- User segmentation must go beyond demographic variables, incorporating psychographic and emotional criteria.
- It is crucial to promote ethics in the use of neuromarketing, especially in contexts where technologies might subconsciously influence consumer behavior.
- Community-generated content, along with emotional narratives, should be considered a strategic asset, not merely a communication tool.

### 5.2 Future lines of research

Based on the identified gaps and emerging trends, the following lines of research are proposed for future studies:

- Longitudinal studies analyzing the sustainability of emotional engagement over time and its relationship with user retention.
- Development of hybrid models that integrate neurophysiological, behavioral, and contextual variables in evaluating marketing campaigns.
- Research in underrepresented regions (Latin America, Africa, Southeast Asia), with special attention to emerging markets and distinct cultural behaviors.
- Advanced applications of artificial intelligence and machine learning for real-time emotional personalization.
- Ethical and regulatory analyses of the use of neurocognitive technologies applied to digital consumption, especially concerning vulnerable audiences (minors, addictive users, etc.).

Based on the presented findings, it is concluded that the intersection of digital marketing, neuroscience, and video games represents not only a strategic opportunity for brands but also fertile ground for the theoretical and methodological development of new research that positions the digital consumer as a central actor in the contemporary cultural ecosystem.

## Data Availability

All data supporting the conclusions of this review are contained within the article and its Supplementary material. The complete search log, screening records and study-level extraction sheet are available from the corresponding author on request.

## Supplementary material

Supplementary Appendix S1 Search strategy. Databases searched with dates and per-query yields, all thirteen Boolean strings as executed, the deduplication procedure, the screening cascade, and inter-rater reliability statistics.

Supplementary Table 2 Characteristics of the 54 included studies. Title, authors, methodological design, sample size, focus of findings, country, risk-of-bias rating, year of publication and identification route for each study.

## References

[B1] Abbas Q. (2024). The impact of personalization strategies on consumer engagement and conversion rates in digital marketing. Int. J. Adv. Multidiscip. Res. Stud. 4, 452–454.

[B2] Adjust (2025). The Gaming App Insights Report: 2025 Edition. Berlin: Adjust GmbH. Available online at: https://www.adjust.com/blog/gaming-app-insights-2025/ (Accessed August 27, 2026).

[B3] Argyris Y. A. Wang Z. Kim Y. Yin Z. (2020). The effects of visual congruence on increasing consumers' brand engagement: an empirical investigation of influencer marketing on Instagram using deep-learning algorithms for automatic image classification. Comput. Human Behav. 112:106443. doi: 10.1016/j.chb.2020.106443

[B4] Arora A. Bansal S. Kandpal C. Aswani R. Dwivedi Y. (2019). Measuring social media influencer index: insights from Facebook, Twitter and Instagram. J. Retail. Consum. Serv. 49, 86–101. doi: 10.1016/j.jretconser.2019.03.012

[B5] Ascarza E. Netzer O. Runge J. (2025). Personalized game design for improved user retention and monetization in freemium games. Int. J. Res. Market. 42(4, Part A), 975–995. doi: 10.1016/j.ijresmar.2025.01.006

[B6] Behl A. Jayawardena N. Shankar A. Gupta M. Lang L. D. (2024). Gamification and neuromarketing: A unified approach for improving user experience. J. Consum. Behav. 23, 218–228. doi: 10.1002/cb.2178

[B7] Bhardwaj A. Thapa S. B. Gandhi A. (2024). Advances in neuromarketing and improved understanding of consumer behaviour: analysing tool possibilities and research trends. Cogent Bus. Manage. 11:2376773. doi: 10.1080/23311975.2024.2376773

[B8] Bigné E. Andreu L. (2004). Emoción, satisfacción y fidelidad en el contexto del cine. Rev. Esp. Investig. Market. ESIC 8, 5–25.

[B9] Bonadeo M. J. (2005). Odotipo: historia natural del olfato y su función en la identidad de marca (Vol. 3, Colección Investigaciones y Tesis). Universidad Austral, Facultad de Comunicación. Available online at: https://riu.austral.edu.ar/handle/123456789/311 (Accessed April 30, 2025).

[B10] Bonetti F. Perry P. Fernie J. (2016). The evolution of luxury fashion retailing in China. Luxe. Fash. Retail Manage. 49–67. doi: 10.1007/978-981-10-2976-9_4

[B11] Braidot N. (2006). Neuromarketing. Buenos Aires: Granica.

[B12] Carlson J. R. Hanson S. Pancras J. Ross W. T. Jr. Rousseau-Anderson J. (2021). Social media advertising: how online motivations and congruency influence perceptions of trust. J. Consum. Behav. 20, 197–213. doi: 10.1002/cb.1989

[B13] Cenizo C. (2025). A neuromarketing approach to consumer behavior on web platforms. Int. J. Consum. Stud. 49:e70034. doi: 10.1111/ijcs.70034

[B14] Chan A. Astari D. (2017). The analysis of content marketing in online fashion shops in Indonesia. Rev. Integr. Bus. Econ. Res. 6, 225–233. doi: 10.13140/RG.2.2.11591.11683

[B15] Chen X. Qasim H. (2021). Does E-brand experience matter in the consumer market? Explaining the impact of social media marketing activities on consumer-based brand equity and brand love. J. Consum. Behav. 20, 1065–1077. doi: 10.1002/cb.1915

[B16] Chu S. C. Chen H. T. (2019). Impact of consumers' corporate social responsibility-related activities in social media on brand attitude, electronic word-of-mouth intention, and purchase intention: a study of Chinese consumer behavior. J. Consum. Behav. 18, 453–462. doi: 10.1002/cb.1784

[B17] Cuevas-Molano E. Matosas-López L. Bernal-Bravo C. (2021). Factors increasing consumer engagement of branded content in Instagram. IEEE Access 9, 143531–143548. doi: 10.1109/ACCESS.2021.3121186

[B18] Cui Y. Li J. Zhang Y. (2022). The impacts of game experience and fanwork creation on game loyalty: mediation effect of perceived value. Technol. Forecast. Soc. Change 176:121495. doi: 10.1016/j.techfore.2022.121495

[B19] Dubois L. E. Chalk A. (2024). Service withdrawal: the uncertain future of the games-as-a-service model. Convergence. 31, 479–499. doi: 10.1177/13548565241256888

[B20] Edelman. (2019). “2019 Edelman trust barometer special report,”: in Brands We Trust? (New York, NY: Edelman).

[B21] Elias H. Filgueiras E. Carvalho B. J. A. (2015). “Ads-on games and fake brands: Interactions, commercials and playful branding,” in Design, User Experience and Usability. Interactive Experience Design (Libro de actas de DUXU 2015, Vol. III, LNCS 9188), ed. A. Marcus (Cham: Springer), 251–262. doi: 10.1007/978-3-319-20889-3_24

[B22] Fisher C. E. Chin L. Klitzman R. (2010). Defining neuromarketing: practices and professional challenges. Harv. Rev. Psychiatry 18, 230–237. doi: 10.3109/10673229.2010.496623 20597593 PMC3152487

[B23] Fletcher K. A. Gbadamosi A. (2022). Examining social media live stream's influence on the consumer decision-making: a thematic analysis. Electron. Commer. Res. 24, 2175–2205. doi: 10.1007/s10660-022-09623-y

[B24] García Galicia J. M. (2010). “Market,” in Los *Secretos del Marketing*. Guatemala: Peter Jordan Consultores.

[B25] Ge J. J. Sui Y. Zhou X. Li G. (2021). Effect of short video ads on sales through social media: the role of advertisement content generators. Int. J. Advert. 40, 1–27. doi: 10.1080/02650487.2020.1848986

[B26] Ghosh T. Sreejesh S. Dwivedi Y. K. (2022a). Brand logos versus brand names: a comparison of the memory effects of textual and pictorial brand elements placed in computer games. J. Bus. Res. 147, 222–235. doi: 10.1016/j.jbusres.2022.04.017

[B27] Ghosh T. Sreejesh S. Dwivedi Y. K. (2022b). Brands in a game or a game for brands? Comparing the persuasive effectiveness of in-game advertising and advergames. Psychol. Market. 39, 2328–2348. doi: 10.1002/mar.21752

[B28] Gupta R. Kapoor A. P. Verma H. V. (2025). Neuro-insights: a systematic review of neuromarketing perspectives across consumer buying stages. Front. Neuroergon. 6:1542847. doi: 10.3389/fnrgo.2025.1542847 40735149 PMC12305819

[B29] Hinsch C. Sheldon K. M. (2013). The impact of frequent social Internet consumption: Increased procrastination and lower life satisfaction. J. Consum. Behav. 12, 496–505. doi: 10.1002/cb.1453

[B30] Hofmann V. Schwayer L. M. Stokburger-Sauer N. E. Wanisch A. T. (2021). Consumers' self-construal: measurement and relevance for social media communication success. J. Consum. Behav. 20, 642–661. doi: 10.1002/cb.1927

[B31] Hu H. Zhang D. Wang C. J. (2019). Impact of social media influencers' endorsement on application adoption: a trust-transfer perspective. Soc. Behav. Personal. 47, 1–12. doi: 10.2224/sbp.8518

[B32] Huang Y. T. Gong A.-D. (2024). The role of game involvement on attention to ads: exploring influencing factors of visual attention to game ads on Instagram Stories. Behav. Inform. Technol. 2024:3706590. doi: 10.1155/2024/3706590

[B33] Kallio K. P. Mäyrä F. Kaipainen K. (2011). At least nine ways to play: approaching gamer mentalities. Games Cult. 6, 327–353. doi: 10.1177/1555412010391089

[B34] Karpenka L. Rudiene E. Morkunas M. Volkov A. (2021). The influence of a brand's visual content on consumer trust in social media community groups. J. Theor. Appl. Electron. Commer. Res. 16, 2424–2441. doi: 10.3390/jtaer16060133

[B35] Keller K. L. Kotler P. (2006). Marketing Management, 12th Edn. Upper Saddle River, NJ: Pearson.

[B36] Kemppainen T. Paananen T. E. (2024). Dualities of digital services: everyday digital services as positive and negative contributors to customer well-being. J. Serv. Theory Pract. 34, 464–490. doi: 10.1108/JSTP-03-2023-0075

[B37] Kim N. Lee H. (2021). Assessing consumer attention and arousal using eye-tracking technology in virtual retail environment. Front. Psychol. 12:665658. doi: 10.3389/fpsyg.2021.665658 34434136 PMC8380820

[B38] Kotler P. Armstrong G. (2004). Principles of Marketing, 10th Edn. Upper Saddle River, NJ: Pearson Prentice Hall.

[B39] Larios-Gómez E. Fischer L. (2024). Impact of commercial advertisement from the attitude and content of YouTubers. Contad. Adm. 69(2 Especial Mercadotecnia), 120–138. doi: 10.22201/fca.24488410e.2024.5192

[B40] Lee C. T. Hsieh S. H. (2022). Can social media-based brand communities build brand relationships? Examining the effect of community engagement on brand love. J. Theor. Appl. Electron. Commer. Res. 17, 1270–1285. doi: 10.3390/jtaer17060090

[B41] Lee S. Y. Lee J. Ahn H. Moon J. (2019). How implicit mindset influences consumers' perception of company engagement with product complaints online. Soc. Behav. Pers. 47, 1–9. doi: 10.2224/sbp.8451

[B42] Levitt T. (1975). Marketing myopia. Harv. Bus. Rev. 53, 26–47. *(Reprinted in* 2011).

[B43] Liu-Thompkins Y. Okazaki S. Li H. (2022). Artificial empathy in marketing interactions: bridging the human-AI gap in affective and social customer experience. J. Acad. Mark Sci. 50, 1198–1218. doi: 10.1007/s11747-022-00892-5

[B44] Lou C. Kim H. K. (2019). Fancying the new rich and famous? Explicating the roles of influencer content, credibility, and parental mediation in adolescents' parasocial relationship, materialism, and purchase intentions. Front. Psychol. 10:2567. doi: 10.3389/fpsyg.2019.02567 31803110 PMC6872518

[B45] Macey J. Tyrväinen V. Pirkkalainen H. Hamari J. (2022). Does esports spectating influence game consumption? Behav. Inform. Technol. 41, 181–197. doi: 10.1080/0144929X.2020.1797876

[B46] Malfitano Cayuela O. R. (2007). Neuromarketing: Cerebrando Negocios y Servicios. Buenos Aires: Ediciones Granica.

[B47] Martínez-Maldonado A. (2025). From pixels to choices: understanding the influence of video games on decision-making skills. Entertain. Comput. 54:100953. doi: 10.1016/j.entcom.2025.100953

[B48] Mendoza L. E. Marius A. Pérez M. Grimán A. C. (2007). Critical success factors for a customer relationship management strategy. Inf. Softw. Technol. 49, 913–945. doi: 10.1016/j.infsof.2006.10.003

[B49] Moher D. Liberati A. Tetzlaff J. Altman D. G. PRISMA Group (2009). Preferred reporting items for systematic reviews and meta-analyses (PRISMA) statement. PLoS Med. 6:e1000097. doi: 10.1371/journal.pmed.1000097 21603045 PMC3090117

[B50] Murphy E. R. Illes J. Reiner P. B. (2008). Neuroethics of neuromarketing. J. Consum. Behav. 7, 293–302. doi: 10.1002/cb.252

[B51] Naeem M. (2019). Do social networking platforms promote service quality and purchase intention of customers of service-providing organizations? J. Manage. Dev. 38, 561–581 doi: 10.1108/JMD-11-2018-0327

[B52] Newzoo (2023). Global Games Market Report 2023 (Free Version). Amsterdam: Newzoo.

[B53] Nikolinakou A. Phua J. (2019). Do human values matter for promoting brands on social media? How social media users' values influence valuable brand-related activities such as sharing, content creation, and reviews. J. Consum. Behav. 19, 664–675. doi: 10.1002/cb.1790

[B54] Ohme R. Reykowska D. Wiener D. Choromanska A. (2009). Analysis of neurophysiological reactions to advertising stimuli by means of EEG and galvanic skin response measures. J. Neurosci. Psychol. Econ. 2, 21–31. doi: 10.1037/a0015462

[B55] Page M. J. McKenzie J. E. Bossuyt P. M. Boutron I. Hoffmann T. C. Mulrow C. D. . (2021). The PRISMA 2020 statement: an updated guideline for reporting systematic reviews. BMJ 372:n71. doi: 10.1136/bmj.n71 33782057 PMC8005924

[B56] Pehlivan E. Sarican F. Berthon P. (2011). Mining messages: exploring consumer response to consumer- vs. firm-generated ads. J. Consum. Behav. 10, 313–321. doi: 10.1002/cb.379

[B57] Prehn-Kristensen A. Göder R. Chirobeja S. Bressmann I. Ferstl R. Baving L. (2009). Sleep in children enhances preferentially emotional declarative but not procedural memories. J. Exp. Child Psychol. 104, 132–139. doi: 10.1016/j.jecp.2009.01.005 19251274

[B58] Puriwat W. Tripopsakul S. (2021). The role of viral marketing in social media on brand recognition and preference. Emerg. Sci. J. 5, 855–867. doi: 10.28991/esj-2021-01315

[B59] Rawnaque F. S. Rahman K. M. Anwar S. F. Vaidyanathan R. Chau T. Sarker F. . (2020). Technological advancements and opportunities in neuromarketing: a systematic review. Brain Inform 7:10. doi: 10.1186/s40708-020-00109-0 32955675 PMC7505913

[B60] Rizun N. Kucharska W. (2018). “Text mining algorithms for extracting brand knowledge: the fashion industry case,” in Innovation Management and Education Excellence through Vision 2020: Proceedings of the 31st International Business Information Management Association Conference (IBIMA), ed. K. S. Soliman (Milan: International Business Information Management Association (IBIMA)), 1972–1983. doi: 10.2139/ssrn.3148476

[B61] Rodríguez-Ramírez R. Arbaiza F. Yalán E. (2021). Perception of UPC university gamers on the adidas in-game advertising in the ‘FIFA20′ videogame. Index Comun. 11, 21–40. doi: 10.33732/ixc/11/02Percep

[B62] Saura J. R. Palos-Sanchez P. Rodríguez Herráez B. (2020). Digital marketing for sustainable growth: business models and online campaigns using sustainable strategies. Sustainability 12:1003. doi: 10.3390/su12031003

[B63] Saveljeva J. Volkova T. (2025). A survey on digital trust: towards a validated definition. Digital 5:14. doi: 10.3390/digital5020014

[B64] Sesar V. Hunjet A. Martinčević I. (2023). Generation Z purchase intentions: does sponsorship disclosure matter? Bus. Syst. Res. 14, 158–172. doi: 10.2478/bsrj-2023-0017

[B65] Shin D.-H. (2013). User experience in social commerce: in friends we trust. Behav. Inform. Technol. 32, 52–67. doi: 10.1080/0144929X.2012.692167

[B66] Souki G. Q. Chinelato F. B. Gonçalves Filho C. (2022). Sharing is entertaining: the impact of consumer values on video sharing and brand equity. J. Res. Interact. Market. 16, 118–136. doi: 10.1108/JRIM-03-2020-0057

[B67] Strasburger V. C. Hogan M. J. Mulligan D. A. . (2013). Children, adolescents, and the media. Pediatrics 132, 958–961. doi: 10.1542/peds.2013-2656 28448255

[B68] Teng C. I. Huang T. L. Huang G. L. Wu C. N. Cheng T. C. E. Liao G. Y. (2024). Creatability, achievability, and immersibility: new game design elements that increase online game usage. Int. J. Inf. Manage. 75:102732. doi: 10.1016/j.ijinfomgt.2023.102732

[B69] Teo L. X. Leng H. K. Phua Y. X. P. (2019). Marketing on Instagram: social influence and image quality on perception of quality and purchase intention. Int. J. Sports Mark. Sponsors. 20, 321–332. doi: 10.1108/IJSMS-04-2018-0028

[B70] Toh W. (2021). The economics of decision-making in video games. Game Stud. 21. Available online at: https://gamestudies.org/2103/articles/toh

[B71] Torres P. Augusto M. Matos M. (2019). Antecedents and outcomes of digital influencer endorsement: an exploratory study. Psychol. Market. 36, 1267–1276. doi: 10.1002/mar.21274

[B72] Upadhyay Y. Paul J. Baber R. (2022). Effect of online social media marketing efforts on customer response. J. Consum. Behav. 21, 554–571. doi: 10.1002/cb.2031

[B73] Wang T. Yeh R. K. J. Yen D. C. (2015). Influence of customer identification on online usage and purchasing behaviors in social commerce. Inform. Syst. Manage. 31, 805–814. doi: 10.1080/10447318.2015.1067481

[B74] Wong A. (2021). The nature of peer-initiated brand communities on social media platforms. J Consum. Behav 20, 1629–1647. doi: 10.1002/cb.1978

[B75] Ye G. Guan X. Hudders L. (2025). A meta-analysis of the antecedents and consequences of advertising value. J. Advert. doi: 10.1080/00913367.2024.2309923

[B76] Yucra-Quispe L. M. Espinoza-Montoya C. Núñez-Pacheco R. Aguaded I. (2022). From consumers to prosumers: the transmedia storytelling in two mobile games for adolescents and young people. Rev. Comun. 21, 433–450. doi: 10.26441/RC21.1-2022-A22

[B77] Zagni L. M. Baima G. Do B. . (2025). Convergence and divergence in digital game-based advertising: a systematic review of advergames and in-game advertising. Rev. Manag. Sci. 20, 511–534. doi: 10.1007/s11846-025-00876-z

[B78] Zetian D. Xue J. Du J. Tan W. H. (2024). Unlocking play willingness: the dual pathways of curiosity drive and downward social comparison in game advertising. Front. Psychol. 15:1374649. doi: 10.3389/fpsyg.2024.1374649 39246312 PMC11378734

[B79] Zhang Y. Choi H. K. (2023). Brand anthropomorphism and consumer brand evangelism on social networking sites: prevention focus as a moderator. Soc. Behav. Personal. 51:e12726. doi: 10.2224/sbp.12726

[B80] Zhao M. Thadani D. R. (2021). Investigating deception and the impacts on online game players' choice in China. Glob. Media China 6, 416–442. doi: 10.1177/20594364211027284

[B81] Zhao Z. Guo Y. Liu Y. Xia H. Lan H. (2022). The effect of image–text matching on consumer engagement in social media advertising. Soc. Behav. Pers. 50, 1–11. doi: 10.2224/sbp.11799

