# Supplementary material for: Neuromarketing and the gaming consumer: a systematic review of attention, emotion, and memory in player engagement and retention
Source: Front Neuroergon. 2026 Sep 10;7:1918889. doi: 10.3389/fnrgo.2026.1918889 (PMC13601311; doi:10.3389/fnrgo.2026.1918889)
Supplement: Supplementary file 1 [file Data_Sheet_1.docx]

Supplementary Material

# 1 Supplementary Figures and Tables

**Table 2. Characteristics of the 54 included studies. The Identification route column distinguishes studies retrieved through database searching from those identified through citation tracking.**

| **Title of the article (subtitle may be cropped from the full title in order to improve readability)** | **Authors** | **Methodological design** | **Sample size (n)** | **Focus of the results and findings** | **Country** | **Risk of bias** | **Year** | **Identification route** |
| --- | --- | --- | --- | --- | --- | --- | --- | --- |
| Analysis of neurophysiological reactions to advertising stimuli by means of EEG and galvanic skin response measures | Ohme, R. et al. | Physiological Measurement | 64 | Emotional Activation & Recall | Poland | Low | 2009 | Database searching |
| Mining messages: Exploring consumer response to consumer- vs. firm-generated ads. | Pehlivan, E.  Sarican, F.  Berthon, P. | Content / Narrative Analysis | 75 | User-Generated Content & Advertising Themes | USA | Moderate | 2011 | Database searching |
| User experience in social commerce: in friends we trust. | Shin, D-H. | Multi-phase / Mixed-method Survey | 329 | S-commerce User Intentions | USA | Low | 2013 | Database searching |
| The impact of frequent social Internet consumption: Increased procrastination and lower life satisfaction. | Hinsch, C  Sheldon, KM. | Field / Longitudinal Experiment | 143 | Social Internet Usage & Well-being | USA | High | 2013 | Database searching |
| Children, Adolescents, and the Media. | Strasburger, V. C. et al. | Policy Statements | N/A | Media Use & Health Risks in Youth | USA | Moderate | 2013 | Database searching |
| Ads-on games and fake brands: Interactions, commercials and playful branding | Elias H. Filgueiras E. Carvalho B. | Content / Narrative Analysis | 24 | Brand Immersion & Credibility | Portugal | Moderate | 2015 | Database searching |
| Influence of Customer Identification on Online Usage and Purchasing Behaviors in Social Commerce. | Wang, T.  Yeh, R.  Yen, D. | Online / Offline Survey | 242 | Customer Loyalty & Purchase Behavior Drivers | Taiwan | Low | 2015 | Database searching |
| The analysis of content marketing in online fashion shops in Indonesia | Chan, A.  Astari, D. | Multi-phase / Mixed-method Survey | 100 | Personalized Content & Loyalty | Indonesia | High | 2017 | Database searching |
| Text mining algorithms for extracting brand knowledge: The fashion industry case | Rizun, N. Kucharska, W. | Content / Narrative Analysis | N/A | Text Analysis for Brand Attributes | Poland | High | 2018 | Database searching |
| Do social networking platforms promote service quality and purchase intention of customers of service-providing organizations? | Naeem, M. | Online / Offline Survey | 387 | Consumer Confidence & Brand Engagement | UK | Low | 2019 | Database searching |
| Marketing on Instagram Social influence and image quality on perception of quality and purchase intention | Teo, LX. Leng, HK. Phua, YXP. | Lab / Controlled Experiment | 99 | Perceived Quality & Purchase Intention | Taiwan | Low | 2019 | Database searching |
| Measuring social-media influencer index: Insights from Facebook, Twitter and Instagram | Arora, N. et al. | Content / Narrative Analysis | >300 | Influencer Impact Prediction | India | High | 2019 | Database searching |
| How implicit mindset influences consumers’ perception of company engagement with product complaints online. | Sang Yeal Lee, et al. | Lab / Controlled Experiment | 356 | Company Engagement & Implicit Mindset | USA | Low | 2019 | Database searching |
| Impact of social media influencers’ endorsement on application adoption: A trust transfer perspective. | Hu H.  Dandan Z.  Chengjun W. | Online / Offline Survey | 371 | App Adoption & Trust Influencers | China | Low | 2019 | Database searching |
| Impact of consumers' corporate social responsibility‐related activities in social media on brand attitude, electronic word‐of‐mouth intention, and purchase intention: A study of Chinese consumer behavior. | Chu, S-C.  Chen, H-T. | Online / Offline Survey | 631 | CSR Activities & Consumer Behavior Outcomes | China | Low | 2019 | Database searching |
| Explicating the Roles of Influencer Content, Credibility, and Parental Mediation in Adolescents’ Parasocial Relationship, Materialism, and Purchase Intentions | Lou C.  Kim H.K. | Online / Offline Survey | 500 | Influencer Content & Adolescent Behavior | Singapore | Low | 2019 | Database searching |
| How social media users' values influence valuable brand‐related activities such as sharing, content creation, and reviews | Nikolinakou, A. Phua, J. | Online / Offline Survey | 491 | Values & Social Media Brand Activities | USA | Low | 2019 | Database searching |
| Antecedents and outcomes of digital influencer endorsement: An exploratory study | Torres, P.  Augusto, M.  Matos, M. | Multi-phase / Mixed-method Survey | >210 | Influencer Credibility & Consumer Trust | Portugal | High | 2019 | Database searching |
| The effects of visual congruence on increasing consumers' brand engagement | Argyris, YA et al. | Field / Longitudinal Experiment | N/A | Visual Congruence & Influencer/Brand Engagement | USA | High | 2020 | Database searching |
| Technological advancements and opportunities in neuromarketing: A systematic review | Rawnaque, F. S. et al. | Systematic Review | >60 | Top Applied Tools (EEG, Eye Tracking, Facial Recognition) | Bangladesh | High | 2020 | Database searching |
| Digital marketing for sustainable growth: Business models and online campaigns using sustainable strategies | Saura, J. R. et al. | Case Studies | >150 | Sustainability as Emotional Value | Spain | High | 2020 | Database searching |
| Factors Increasing Consumer Engagement of Branded Content in Instagram | Cuevas-Molano, E. Matosas-López, L. Bernal-Bravo, C. | Content / Narrative Analysis | 680 | Social Media Engagement Factors | Spain | Moderate | 2021 | Database searching |
| Investigating deception and the impacts on online game players' choice in China | Zhao, MJ. Thadani, DR. | Online / Offline Survey | 356 | Player Motivation & Game Strategy | China | Low | 2021 | Database searching |
| The nature of peer‐initiated brand communities on social media platforms. | Wong, A. | Online / Offline Survey | 205 | Brand Community Engagement & Loyalty | Singapore | Low | 2021 | Database searching |
| Social media advertising: How online motivations and congruency influence perceptions of trust. | Carlson, JR. et al. | Lab / Controlled Experiment | 409 | Trust in Social Media Advertising & Motivations | USA | Low | 2021 | Database searching |
| Assessing consumer attention and arousal using eye-tracking technology in virtual retail environment | Kim, N.  Lee, H. | Physiological Measurement | 50 recruited; 46 with valid gaze data | Visual Attention & Emotional Arousal | South Korea | Low | 2021 | Database searching |
| The role of viral marketing in social media on brand recognition and preference | Puriwat, W. Tripopsakul, S. | Online / Offline Survey | 360 | Viral Marketing & Brand Preference | Thailand | High | 2021 | Database searching |
| Effect of short video ads on sales through social media: the role of advertisement content generators | Ge, JJ. et al. | Field / Longitudinal Experiment | 260 | Short Video Ads & Product Sales | China | High | 2021 | Database searching |
| Perception of upc university gamers on the adidas in-game advertising in the ‘fifa20’ videogame | Rodríguez-Ramírez R.  Arbaiza F.  Yalán E. | Interviews | 18 | In-Game Advertising Perception & Impact | Peru | Moderate | 2021 | Database searching |
| Consumers' self‐construal: Measurement and relevance for social media communication success. | Hofmann, Verena, et al. | Lab / Controlled Experiment | 143 | Self-Construal & Social Media Response | Austria | Low | 2021 | Database searching |
| The Influence of a Brand's Visual Content on Consumer Trust in Social Media Community Groups | Karpenka, L. et al. | Online / Offline Survey | 387 | Consumer Confidence & Brand Engagement | Lithuania | Low | 2021 | Database searching |
| Does E‐Brand experience matter in the consumer market? Explaining the impact of social media marketing activities on consumer‐based brand equity and love. | Chen, X.  Qasim, H. | Online / Offline Survey | 441 | Social Media Marketing & Brand Love/Equity | China | High | 2021 | Database searching |
| Does esports spectating influence game consumption? | Macey, J. et al. | Online / Offline Survey | 194 | Esports Watching/Gaming/Purchasing Intentions | Finland | Low | 2022 | Database searching |
| Sharing is entertaining: the impact of consumer values on video sharing and brand equity | Souki, GQ. Chinelato, FB. Filho, CG. | Online / Offline Survey | 368 | Brand Attachment & Equity Drivers | Portugal | Low | 2022 | Database searching |
| From consumers to prosumers: the transmedia storytelling in two mobile games for adolescents and young people | Yucra-Quispe, LM et al. | Case Studies | N/A | Transmedia Storytelling & User Behavior | Peru | Moderate | 2022 | Database searching |
| Can social media-based brand communities build brand relationships? Examining the effect of community engagement on brand love. | Lee, C.  Hsieh, S. | Online / Offline Survey | 360 | Community Engagement & Brand Love | China | Low | 2022 | Database searching |
| Examining social media live stream's influence on the consumer decision-making: a thematic analysis | Fletcher, KA. Gbadamosi, A. | Field / Longitudinal Experiment | 500 | Live Stream Impact on Consumer Behavior | UK | Moderate | 2022 | Database searching |
| Brands in a game or a game for brands? Comparing the persuasive effectiveness of in-game advertising and advergames | Ghosh T.  Sreejesh S.  Dwivedi Y.K. | Lab / Controlled Experiment | 250 | Gamification Format & Brand Attitude/Engagement | India | Low | 2022 | Database searching |
| The impacts of game experience and fanwork creation on game loyalty | Cui, Y. Li, J. Zhang, Y. | Multi-phase / Mixed-method Survey | 305 | Active Participation & Emotional Bond | China | High | 2022 | Database searching |
| The effect of image–text matching on consumer engagement in social media advertising. | Zhao, Z et al. | Content / Narrative Analysis | 322 | Image-Text Coherence & Consumer Engagement | China | High | 2022 | Database searching |
| Effect of online social media marketing efforts on customer response. | Upadhyay, Y.  Paul, J.  Baber, R. | Online / Offline Survey | 318 | Brand Equity & Customer Response | India | High | 2022 | Database searching |
| Brand logos versus brand names: A comparison of the memory effects of textual and pictorial brand elements placed in computer games | Ghosh T.  Sreejesh S.  Dwivedi Y.K. | Lab / Controlled Experiment | 180 | Attention & Brand Memory in Advertising | India | Low | 2022 | Database searching |
| Generation Z Purchase Intentions: Does Sponsorship Disclosure Matter? | Sesar, V. Hunjet, A. Martincevic, I. | Online / Offline Survey | 364 | Sponsorship Disclosure & Purchase Intention | Croatia | High | 2023 | Database searching |
| Brand anthropomorphism and consumer brand evangelism on social networking sites: Prevention focus as a moderator. | Zhang, Y.  Choi, H. | Lab / Controlled Experiment | 366 | Brand Anthropomorphism & Evangelism | China | Low | 2023 | Database searching |
| The Role of Game Involvement on Attention to Ads: Exploring Influencing Factors of Visual Attention to Game Ads on Instagram Stories | Huang Y.-T. Gong A.-D. | Lab / Controlled Experiment | 80 | Ad Effectiveness & Attention | Taiwan | Low | 2024 | Database searching |
| Impact of commercial advertisement from the attitude and content of YouTubers | Larios-Gómez E.  Fischer L. | Descriptive / Non-Experimental | 1536 | YouTuber Influence & Ad Impact | Mexico | Low | 2024 | Database searching |
| Unlocking play willingness: the dual pathways of curiosity drive and downward social comparison in game advertising | Zetian D. et al. | Lab / Controlled Experiment | 1300 | Missing Information & Play Willingness | China | Low | 2024 | Database searching |
| Dualities of digital services: everyday digital services as positive and negative contributors to customer well-being | Kemppainen T.  Paananen T.E. | Interviews | 14 | Digital Services & Well-being (Dualities) | Finland | Moderate | 2024 | Database searching |
| Service withdrawal: The uncertain future of the games-as-a-service model | Dubois L.-E.  Chalk A. | Case Studies | 5 | Game-as-a-Service (GaaS) Termination Factors | Canada | Moderate | 2024 | Database searching |
| Creatability, achievability, and immersibility: New game design elements that increase online game usage | Teng C.-I. et al. | Field / Longitudinal Experiment | 546 | Game Continuance & Player Satisfaction | Taiwan | Low | 2024 | Database searching |
| Advances in neuromarketing and improved understanding of consumer behaviour: Analysing tool possibilities and research trends | Bhardwaj, A.  Thapa, S. B.  Gandhi, A. | Systematic Review | N/A | Combined Technologies for Analysis | India | High | 2024 | Database searching |
| Gamification and neuromarketing: A unified approach for improving user experience | Behl, A., Jayawardena, N., Shankar, A., Gupta, M., & Lang, L. D. | Online / Offline Survey | 267 responses (1,278 firms contacted) | Marketers' perceptions of gamification and neuromarketing; Self-Determination Theory framework | India | Low | 2024 | Citation tracking |
| A Meta-Analysis of the Antecedents and Consequences of Advertising Value | Ye G. et al. | Meta-Analysis | 98 | Advertising value drivers & Credibility and entertainment matter | Belgium | High | 2025 | Database searching |
| Personalized game design for improved user retention and monetization in freemium games | Ascarza, E., Netzer, O., & Runge, J. | Field / Longitudinal Experiment | 329,999 users; approx. 79 million rounds | Personalised difficulty adjustment; engagement, retention and monetisation in a free-to-play game | USA | Low | 2025 | Citation tracking |

# 2 Supplementary Appendix S1: Search Strategy

This appendix documents the identification of studies for the present review. Records were identified through two complementary routes: (a) systematic Boolean searching of three bibliographic databases, and (b) purposive and citation-based identification of conceptual and neuromarketing literature not retrievable through the database queries. Both routes are reported in full below.

## 2.1 Databases searched and records retrieved

| **Database** | **Platform / segment** | **Queries** | **Records retrieved** | **Date searched** |
| --- | --- | --- | --- | --- |
| Web of Science | Web of Science Core Collection (ALL fields) | 5 | 381 | 29 April 2025 |
| Scopus | Elsevier Scopus (TITLE-ABS-KEY) | 4 | 297 | 29 April 2025 |
| Psychology and Behavioral Sciences Collection | EBSCOhost (db = pbh) | 4 | 654 | 2 May 2025 |
| TOTAL RECORDS RETRIEVED | - | 13 | 1,332 | - |
| Duplicates removed | - | - | 304 | - |
| TOTAL AFTER DEDUPLICATION | - | - | 1,028 | - |

## 2.2 Search strings as executed

Strings are reproduced verbatim from the search log. Yields are the raw record counts returned at the time of searching.

Web of Science — Core Collection, ALL fields (381 records)

WoS-1 — 87 records

ALL=((audiovisual OR video OR add) AND consumer AND Media AND Instagram)

WoS-2 — 36 records

ALL=((audiovisual OR video OR add) AND (consumer OR customer) AND Media AND product AND Instagram)

WoS-3 — 119 records

ALL=((audiovisual OR video OR add) AND (consumer OR customer) AND (social media OR Instagram) AND video game)

WoS-4 — 14 records

ALL=((audiovisual OR video OR reel) AND (consumer OR customer OR prosumer) AND (social media OR Instagram) AND video game AND (advertisement OR add))

WoS-5 — 125 records

ALL=((audiovisual OR video OR reel) AND (consumer OR customer OR prosumer) AND (social media OR Instagram) AND (advertisement OR add))

Scopus (297 records)

SCO-1 — 177 records

( "consumer" OR "customer" OR "prosumer" ) AND ( "social media" OR "instagram" ) AND ( "audiovisual" OR "video" OR "reel" ) AND ( "advertisement" OR "add" ) AND videogame

SCO-2 — 57 records

( "consumer" OR "customer" OR "prosumer" ) AND ( "instagram" ) AND ( "audiovisual" OR "video" OR "reel" ) AND ( "advertisement" OR "add" ) AND videogame

SCO-3 — 56 records

( "consumer" ) AND ( "instagram" ) AND ( "audiovisual" OR "video" OR "reel" ) AND ( "advertisement" OR "add" ) AND videogame

SCO-4 — 7 records

( "consumer" ) AND ( "instagram" ) AND ( "video" ) AND ( "add" ) AND videogame

Psychology and Behavioral Sciences Collection — EBSCOhost (654 records)

PBSC-1 — 3 records

consumer AND social media AND advertisement AND video

PBSC-2 — 3 records

consumer AND social media AND commercial AND video

PBSC-3 — 615 records

consumer AND social media OR instagram AND commercial AND video

PBSC-4 — 33 records

consumer AND social media AND commercial AND videogame

**Note on PBSC-3.** This string was entered without parentheses around the OR operator. Under EBSCOhost operator precedence it is evaluated as (consumer AND social media) OR (instagram AND commercial AND video), which is broader than the intended conjunction and accounts for the disproportionate yield of this single query (615 of 1,332 records, 46.2%). The resulting records passed through the same screening criteria as all others, and the great majority were excluded at title-and-abstract stage. This is reported here for transparency and is acknowledged as a limitation of the search.

## 2.3 Supplementary identification

The database queries above target consumer, social-media, audiovisual, advertising, and video-game vocabulary. They do not contain neuromarketing or psychophysiological method terms, and consequently returned very few records in that literature: of the 1,028 deduplicated records, only one mentions neuromarketing in its title or abstract, four mention eye-tracking, and one mentions electroencephalography. The conceptual and neuromarketing literature synthesised in this review was therefore identified through a separate, purposive and citation-based route, consistent with the snowballing strategy described in 2.1 of the manuscript.

| **Route** | **Content** | **Records** |
| --- | --- | --- |
| Thematically assembled reference set | Foundational literature on marketing, emotion, sensation, neuromarketing, and experiential marketing (themes: Marketing, MKT+Emociones, Neuromktng, Sensaciones, Experiencial+emocional), including monographs and Spanish-language sources not indexed in the databases searched | 31 |
| Citation tracking and forward-citation search | Additional empirical records identified from reference lists of included studies and via journal alerts, including game-advertising and in-game advertising studies | 27 |
| Subtotal, supplementary identification | — | 58 |

## 2.4 Eligibility limiters

- Languages: English and Spanish.
- Publication period: 2009–2025.
- Document types: peer-reviewed articles, doctoral dissertations, systematic reviews, and empirical studies. Books and book chapters were retained only where they constitute foundational conceptual sources.
- Subject areas: marketing, consumer psychology, applied neuroscience, communication sciences, video games, and technology.
- Mandatory criterion: application of digital marketing techniques in video game contexts OR explicit neuromarketing application, plus at least one further inclusion criterion (see 2.2).

## 2.5 Screening cascade

| **Stage** | **Criterion applied** | **Records** |
| --- | --- | --- |
| Identification | Records retrieved from 3 databases across 13 queries | 1,332 |
| Deduplication | Duplicate records removed across databases | -304 |
| Records screened | Unique records entering screening | 1,028 |
| Screening | Language (English / Spanish) | 1,027 |
| Screening | Accessible DOI | 943 |
| Eligibility | Full text not retrievable | 894 |
| Eligibility | Not addressing marketing–emotions, neuromarketing, or neuroscience | 120 |
| Inclusion | Not addressing video-game consumers or social-media consumers | 52 |
| INCLUDED IN REVIEW | - | 52 |

A further two studies were identified through citation tracking and met the same eligibility criteria, giving 54 included studies.

## 2.6 Record management, deduplication, and reviewer agreement

Bibliographic records retrieved from the three databases were exported in RIS format and imported into Zotero (v7) for reference management. Duplicate records were identified using Zotero’s duplicate-detection function and subsequently verified manually by the reviewers before removal. Of the 1,332 records retrieved, 304 were removed as duplicates, and the final deduplicated library contained 1,028 unique records.

Records were screened by two reviewers, who divided the records between them. To assess inter-rater reliability, a subsample of 220 records (21.4% of the 1,028 unique records) was double-screened at the title-and-abstract stage. Agreement on this subsample was 96.4% (212 of 220 records), with 8 disagreements, giving a Cohen’s kappa of 0.82, which indicates almost perfect agreement beyond chance. Across the full screening process, including full-text assessment, 36 disagreements arose in total; all 36 were resolved by discussion between the two reviewers. The protocol provided for arbitration by a third senior researcher from the same research group had consensus not been reached, but all disagreements were settled by discussion and this provision was not invoked.

Supplementary forward-citation searching was carried out using Google Scholar together with direct journal alerts for the five journals most frequently represented in the initial sample: Journal of Business Research, Psychology & Marketing, Frontiers in Psychology, Computers in Human Behavior, and Journal of Interactive Marketing.

## 2.7 Availability

The complete search log, including per-query result exports, the deduplicated record pool, and the screening and extraction sheets, is available from the authors on request and can be deposited in a public repository (e.g., OSF or Zenodo) on acceptance.

Session-bound result URLs recorded in the search log for Web of Science and Scopus are not permanent and are therefore not reproduced here; the query strings and search dates reported in sections 2.1 and 2.2 of this appendix constitute the reproducible record.
